# Palyosulfonoceramides A and B: Unique Sulfonylated Ceramides from the Brazilian Zoanthids *Palythoa caribaeorum *and *Protopalyhtoa variabilis*

**DOI:** 10.3390/md10122846

**Published:** 2012-12-14

**Authors:** Jose Gustavo L. Almeida, Ana Isabel V. Maia, Diego V. Wilke, Edilberto R. Silveira, Raimundo Braz-Filho, James J. La Clair, Leticia V. Costa-Lotufo, Otília Deusdenia L. Pessoa

**Affiliations:** 1 Department of Organic and Inorganic Chemistry, Federal University of Ceara, Fortaleza, CE, 60021-970, Brazil; E-Mails: gugaufc@gmail.com (J.G.L.A.); isabelvmaia@hotmail.com (A.I.V.M.); edil@ufc.br (E.R.S.); braz@uenf.br (R.B.-F.); opessoa@ufc.br (O.D.L.P.); 2 Department of Physiology and Pharmacology, Federal University of Ceara, Fortaleza, CE, 60430-270, Brazil; E-Mail: diegowilke@gmail.com; 3 Xenobe Research Institute, P.O. Box 3052, San Diego, CA 92163, USA; E-Mail: i@xenobe.org

**Keywords:** zoanthids, *Palythoa caribaeorum*, *Protopalythoa variabilis*, sulfonylated ceramides

## Abstract

The zoanthids *Palythoa caribaeorum *and *Protopalythoa variabilis* are among the most abundant marine species along the Brazilian coast. We now report the isolation and structure elucidation of two unprecedented sulfonylated ceramides, palyosulfonoceramide A (**1**) and palyosulfonoceramide B (**2**) from specimens collected off Brazil’s northeastern coast. The structures of **1** and **2** were established using a combination of NMR analyses, including: evaluation of ^1^H, ^13^C, ^1^H–^1^H COSY, ^1^H–^13^C HSQC, ^1^H–^13^C HMBC, and ^1^H–^15^N HMBC NMR spectra, high-resolution mass spectrometry and chemical degradation. In addition, we also isolated the corresponding known ceramides, *N*-((2*S*,3*R*,4*E*,8*E*)-1,3-dihydroxyoctadeca-4,8-dien-2-yl)-hexadecanamide (**3**) and *N*-((2*S*,3*R*,4*E*)-1,3-dihydroxyoctadeca-4-en-2-yl)-hexadecanamide (**4**), which provided further support for the assignments of **1** and **2**.

## 1. Introduction

Cnidarians are a highly diverse and chemically prolific group with over 11,000 species, including corals, jellyfishes and sea anemones [1,2]. They serve as the second most studied group for the isolation of marine natural products [3], surpassed only by Porifera. Cnidarians contain a remarkable structural and functional metabolic diversity offering both biotechnological and therapeutic applications [2]. The anthozoan orders of Alcyonacea and Gorgonacea are the most abundant and have, to date, yielded the most promising therapeutic leads. On the other hand, the order of Zhoantideahas has been examined only modestly. The majority of the discoveries from this order are related to palytoxin, the most potent non-proteinaceous toxin known. First isolated from *Palythoa toxica *[4], palytoxin has since been found in several other *Palythoa* species [5,6,7].

**Figure 1:**
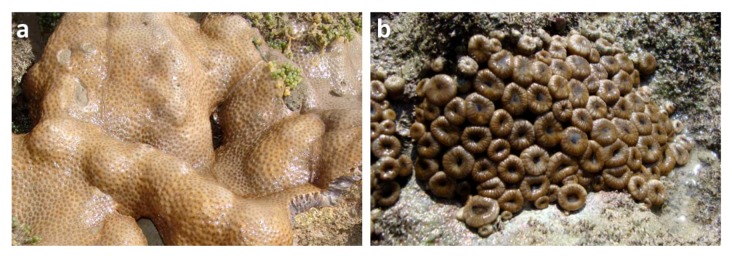
(**a**) *Palythoa caribaeorum* and (**b**) *Protopalythoa variabili*.

Brazil has the second largest coastline of the world. Northeastern Brazil comprises over a half of this coast. Its shores contain a robust diversity of ecosystems including thriving mangrove forests and coral reefs. This region is characterized by a high degree of endemism over different taxonomical groups [8,9]. Its biotechnological potential is robust and has been noted in a series of recent studies [10,11,12,13,14,15]. As part of a program to expand natural product science in Brazil, we were interested in further exploring Zoanthidea, as a potential source for new structural classes. Zoanthids are commonly observed in shallow water communities along the northeastern coast, where *P. caribaeorum* (Duchassaing & Michelotti, 1860) and *P. variabilis* (Duerden 1898) (Figure 1) are often the most prevalent species [16,17]. Previous studies indicated that the genus of *Palythoa* not only serves as the origin of palytoxin but also has been identified as prolific source of steroids, nitrogenated compounds, prostaglandins, glycerol derivatives, and fatty acids [18,19,20,21,22]. Reports on the chemistry of *Protopalythoa *are few, exemplified by the recent isolation of apoptosis-inducing lipidic α-amino acids [14,15]. We now report on the isolation of a novel family of sulfonylated ceramides from extracts of *Palythoa caribaeorum *and *Protopalythoa variabilis *collected off Paracuru Beach in northeastern Brazil.

Lipids, due to their vast chemical characterization, are often overlooked as a source for new structural diversity. While a critical facet of living organisms, the identification of new lipid subclasses remains a key endeavor to unravel discrete metabolic pathways. Sulfur-Containing Lipids (SCLs) is one class that has yet to be fully explored [23,24,25]. The discovery of SCLs is particularly rich dating back to the 1880’s [26] with the identification of sulfate containing lipids and glycolipids, so called sulfolipids [27,28,29]. After a brief perusal of the literature [23,24,25,26,27,28,29,30,31], one quickly gains an admiration for the remarkable positional, oxidative, functional, and stereochemical diversity within SCLs. In a recent and particularly elegant example, the power of modern synthetic chemistry has been applied to gain access to one of the more challenging classes, marine chlorosulfolipids [32,33,34], including undecachlorosulfolipid A [35]. Herein, we describe the isolation of two unprecedented sulfonylated ceramides, designated as palyosulfonoceramide A (**1**) and palyosulfonoceramide B (**2**).

## 2. Results and Discussion

**Figure 2:**
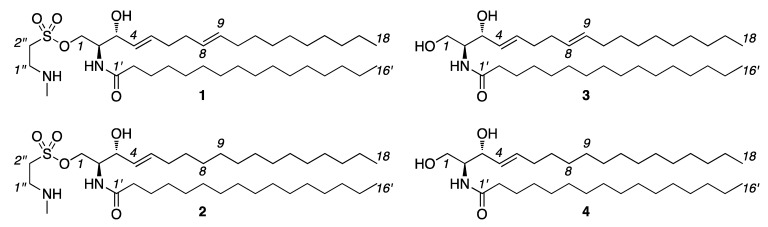
Structures of palyosulfonoceramide A (**1**) and palyosulfonoceramide B (**2**) and associated ceramides **3** and **4** isolated from *Palythoa caribaeorum* and *Protopalythoa variabilis*.

Our approach focused on classical methods of extraction, fractionation, and compound purification (see experimental procedures). We began by evaluating a 21 g crude *n*-hexane extract obtained from 4.7 kg wet weight specimen of *P. caribaeorum*. After fractionation using silica gel chromatography, the most polar fraction, eluted with CH_3_OH, was collected and submitted to a second silica gel purification. Further High Performance Liquid Chromatography (HPLC) chromatographic purification afforded pure palyosulfonoceramide A (**1**) (*t_R_* = 8.0 min) and palyosulfonoceramide B (**2**) (*t_R_* = 9.0 min) along with ceramides, *N*-(2*S*,3*R*,4*E*,8*E*)-1,3-dihydroxyoctadeca-4,8-diene)-hexadecanamide (**3**) and *N*-(2*S*,3*R*,4*E*)-(1,3-dihydroxyoctadeca-4-ene)-hexadecanamide (**4**) (Figure 2). Application of comparable methods to *n*-hexane extracts of *P. variabilis*, also resulted in the isolation of **1**–**4**.

Palyosulfonoceramide A (**1**) was obtained as white amorphous solid, mp 153 °C, and displayed an optical activity, [α]_D_^20^ = +10.2 (*c* 0.053, 4:1 CHCl_3_/MeOH). High-Resolution electrospray ionization mass spectra (HR-ESI-MS) provided a molecular ion [M − H]^−^ at *m/z* 655.5088 compatible with a molecular formula of C_37_H_72_N_2_O_5_S (calculated [M − H]^−^ at *m/z* 655.5084). The infrared spectrum contained bands at 3492–3200 cm^−1^ corresponding to O–H (hydroxyl) and N–H (chelated or free) stretching. Additional absorption bands were observed, indicative of an amide bond including 1627 cm^−1^ (C=O) and 1536 cm^−1^ (C–N) and sulfonyl bonds with 1175 cm^−1^ (SO_2_), 963 cm^−1^ (C–O–S), and 843 cm^−1^ (C–O–S) [36,37,38].

We then turned to NMR analyses (Table 1 and Supporting Information). The ^1^H NMR spectrum of **1** was indicative of a ceramide with a characteristic sphingosine side chain containing two diastereotopic protons at C-1 *δ*_H_ 4.09 and *δ*_H_ 3.91, multiplet for the proton H-2 *δ*_H_ 3.91, NH peak at C-2 *δ*_H_ 7.23, and triplet for the proton H-3 *δ*_H_ 4.02. Coupling constant analyses confirmed a trans-relationship (*J *= 7.3 Hz) between the H-2 and H-3, as commonly observed in ceramides. Alkenic protons at *δ*_H_ 5.68 (dt, *J* = 15.3, 7.1 Hz, H-5) and *δ*_H_ 5.43 (dd, *J* = 15.3, 7.1 Hz, H-4) supported the presence of trans-olefin at C-4 to C-5 with a 15.3 Hz coupling constant (Table 1).

**Table 1:** ^1^H NMR (500 MHz) and ^13^C NMR (125 MHz) data for palyosulfonoceramide A (**1**) and palyosulfonoceramide B (**2**) in 4:1 CDCl_3_/CD_3_OD at 23 °C.

	Palyosulfonoceramide A (1)		Palyosulfonoceramide B (2)	
	^1^H*δ*_H_ (mult, *J* in Hz)	^13^C*δ*_C_ (type)	^1^H*δ*_H_ (mult, *J* in Hz)	^13^C*δ*_C_ (type)
1a	4.09 (m)	64.0 (CH_2_)	3.97 (m)	63.4 (CH_2_)
1b	3.91 (m)		3.74 (m)	
2	3.91 (m)	54.0 (CH)	3.74 (m)	53.9 (CH)
3	4.02 (t, 7.3)	71.4 (CH)	3.88 (t, 7.2)	71.1 (CH)
4	5.43 (dd, 15.3, 7.1)	129.4 (CH)	5.26 (dd, 15.4, 7.1)	128.9 (CH)
5	5.68 (dt, 15.3, 7.1)	133.8 (CH)	5.54 (dt, 14.2, 6.7)	134.3 (CH)
6	2.01 (m)	32.6 (CH_2_)	1.83 (dd, 14.2, 7.0)	32.3 (CH_2_)
7	2.01 (m)	32.4 (CH_2_)	1.08 (m)	31.8 (CH_2_)
8	5.36 (dd, 5.7, 15.9) ^a^	131.1 (CH)	1.08 (m)	29.5–29.1 (CH_2_)
9	5.34 (dd, 5.0, 15.9) ^a^	129.2 (CH)	1.08 (m)	29.5–29.1 (CH_2_)
10	1.91 (q, 6.5)	32.7 (CH_2_)	1.08 (m)	29.5–29.1 (CH_2_)
11–16	1.21 (m)	29.8–29.3 (CH_2_)	1.08 (m)	29.5–29.1 (CH_2_)
17	1.26 (m)	22.7 (CH_2_)	1.08 (m)	22.5 (CH_2_)
18	0.84 (t, 6.9)	14.1 (CH_3_)	0.70 (t, 6.6)	13.8 (CH_3_)
1′		174.3 (C=O)		174.4 (C=O)
2′	2.13 (t, 7.7)	36.6 (CH_2_)	2.01 (t, 7.4)	36.4 (CH_2_)
3′	1.53 (m)	26.0 (CH_2_)	1.41 (m)	25.8 (CH_2_)
4′–14′	1.21 (m)	29.8–29.3 (CH_2_)	1.08 (m)	29.5–29.1 (CH_2_)
15′	1.26 (m)	22.7 (CH_2_)	1.08 (m)	22.5 (CH_2_)
16′	0.84 (t, 6.9)	14.1 (CH_3_)	0.70 (t, 6.6)	13.8 (CH_3_)
1″	3.10 (m)	45.1 (CH_2_)	2.96 (m)	45.3 (CH_2_)
2″	2.01 (m)	32.0 (CH_2_)	1.73 (m)	32.3 (CH_2_)
NCH_3_	2.64 (s)	33.1 (CH_3_)	2.51 (s)	32.7 (CH_3_)
NH	7.23 (d, 8.4)	-	7.32 (d, 8.4)	-
NH	8.96 (br s)	-	8.72 (br s)	-

^a^ Overlap of these peaks thwarted the detection of a coupling constant between H-8 and H-9.

**Figure 3:**
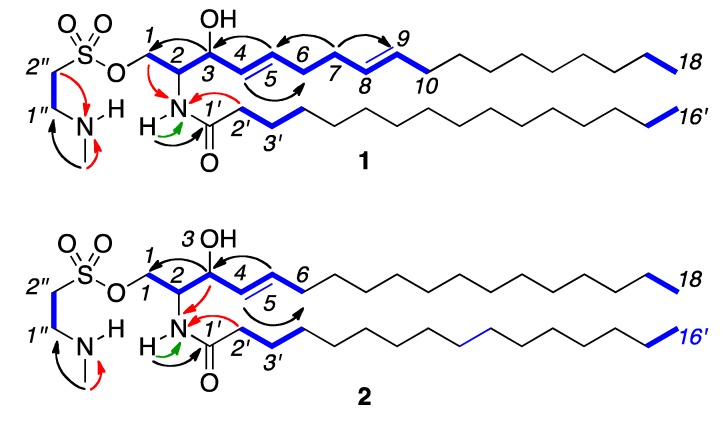
Key ^1^H-^1^H COSY (blue lines), ^1^H-^13^C HMBC (black arrows), ^1^H-^15^N HSQC (green arrows) and ^1^H-^15^N HMBC (red arrows) correlations observed in **1 **and **2**.

**Figure 4:**
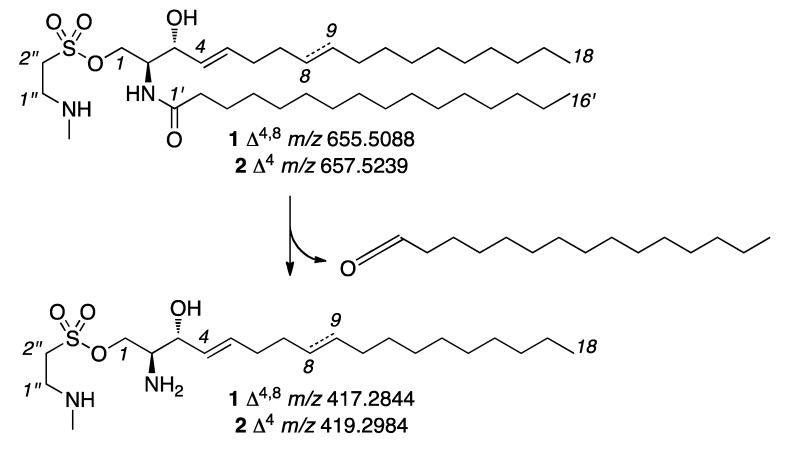
MS/MS fragmentation of **1** and **2**.

We then turned to two-dimensional NMR analyses to further the structure elucidation. The ^1^H-^1^H COSY spectrum identified the connectivity from C-1 to C-10 as well as showing key linkages in the lipid side chain from C-1′ to C-16′ (Figure 3). We then used a combination of Composite Pulse Decoupling (CPD) and Distortionless Enhancement by Polarization Transfer (DEPT) ^13^C NMR along with ^1^H-^13^C HSQC analyses to assign the carbons (Table 1). Evaluation of ^1^H-^13^C HMBC, ^1^H-^15^N HSQC and ^1^H-^15^N HMBC spectra (see Supporting Information) provided further support for the proton and carbon assignments (Table 1) and bond connectivity (Figure 3). The content of the olefin terminus of the sphingosine chain was determined by a combination of high-resolution MS/MS and NMR analysis. MS/MS analysis identified the ejection of C_16_ fatty acid with a daughter ion at *m/z* 417.2844 (Figure 4). Analysis of the NMR data indicated that both the fatty acid and sphingosine chains were linear containing a single methyl terminus at *δ*_H_ 0.84 (t, *J* = 6.6 Hz) for protons H-18 and H-16′, therein confirming that the assignment of the fatty acid unit as palmitic acid. The palmitate amide side chain was further validated through the GC-MS analysis of the methyl ester obtained after methanolysis of **1** with 5% methanolic HCl. The GC chromatogram showed just one peak at *m/z* 270 compatible with the methyl palmitate. 

**Figure 5:**
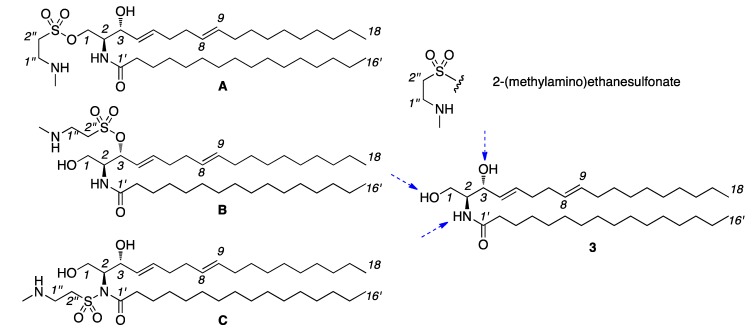
Three sites exist for attachment of the 2-(methylamino)ethanesulfonate to **3** in compound **1** as indicated in isomers **A**–**C**.

Advantageously, we were able to isolate unsulfonylated-ceramide **3** along with **1**. HR-ESI-MS analysis identified a molecular ion [M − H]^−^ for ceramide **3 **at *m/z* 534.4884 compatible with a molecular formula of C_34_H_65_NO_3_ (calculated [M − H]^−^ at *m/z* 534.4886). Comparison of the formula via MS and NMR data (Table 1 *versus*Table 2) between **1** and **3** identified the additional functionality in **1** as C_3_H_7_N_1_SO_2_. Further analysis of the ^1^H NMR spectrum of **3** identified a methyl group at *δ*_H_ 2.64, which was shown by ^1^H-^15^N HMBC analysis to be attached to the remaining nitrogen suggesting the presence of an NHCH_3_ group. In addition, two isolated methylenes were observed at *δ*_H_ 3.10 and *δ*_H_ 2.01, which given the formula, suggested a 2-(methylamino)-ethanesulfonate appendage (Figure 5). Evidence for this group was further supported by the ^1^H to ^13^C and ^1^H to ^15^N correlations observed in both HSQC and HMBC experiments (see Supporting Information). 

**Table 2:** ^1^H NMR (500 MHz) and ^13^C NMR (125 MHz) data for ceramides **3 **and **4** collected in pyridine-*d*_5_ at 23 °C.

	Ceramide 3		Ceramide 4	
	^1^H*δ*_H_ (mult, *J* in Hz)	^13^C*δ*_C_ (type)	^1^H*δ*_H_ (mult, *J* in Hz)	^13^C*δ*_C_ (type)
1a	4.47 (dd, 11.0, 5.3)	62.4 (CH_2_)	4.47 (dd, 11.0, 5.0)	62.5 (CH_2_)
1b	4.30 (dd, 11.0, 5.3)		4.30 (dd, 11.0, 5.0)	
2	4.76 (m)	57.2 (CH)	4.75 (m)	57.2 (CH)
3	4.86 (t, 6.2)	73.6 (CH)	4.86 (t, 6.0)	73.6 (CH)
4	6.07 (dd, 15.4, 6.2)	132.9 (CH)	6.06 (dd, 15.4, 6.3)	132.7 (CH)
5	6.01 (dt, 15.4, 6.2)	131.9 (CH)	5.99 (dt, 15.4, 6.3)	132.6 (CH)
6	2.18 (m)	33.2 (CH_2_)	2.10 (m)	33.0 (CH_2_)
7	2.14 (m)	32.4 (CH_2_)	1.27 (m)	32.4 (CH_2_)
8	5.50 (m)	131.4 (CH)	1.27 (m)	29.9–30.3 (CH_2_)
9	5.50 (m)	130.2 (CH)	1.27 (m)	29.9–30.3 (CH_2_)
10	2.02 (m)	33.2 (CH_2_)	1.27 (m)	29.9–30.3 (CH_2_)
11–16	1.27 (m)	29.8–30.3 (CH_2_)	1.27 (m)	29.9–30.3 (CH_2_)
17	1.27 (m)	23.2 (CH_2_)	1.27 (m)	23.2 (CH_2_)
18	0.87 (t, 5.0)	14.6 (CH_3_)	0.88 (t, 5.8)	14.6 (CH_3_)
1′	-	173.9 (C=O)	-	173.9 (C=O)
2′	2.48 (t, 7.7)	37.2 (CH_2_)	2.47 (t, 7.3)	37.2(CH_2_)
3′	1.84 (m)	26.7 (CH_2_)	1.84 (m)	26.7(CH_2_)
4′–14′	1.27 (m)	29.8–30.3 (CH_2_)	1.27 (m)	29.9–30.3(CH_2_)
15′	1.27 (m)	23.2 (CH_2_)	1.27 (m)	23.2(CH_2_)
16′	0.87 (t, 5.0)	14.6 (CH_3_)	0.88 (t, 5.8)	14.6 (CH_3_)
N–H	8.40 (d, 8.5)	-	8.37 (d, 8.4)	-

At this stage, three items remained unsolved, namely: identification of the position of attachment of the C_3_H_7_N_1_SO_2_ functionality, elucidation of the geometry of the C-8 to C-9 olefin, and validation of the absolute stereochemistry. We began with the positional assignment. As shown in Figure 5, three possibilities exist as given by linkage to the C-1 hydroxyl, C-3 hydroxyl or C-2 amine. We were able to eliminate **C** (Figure 5) due to the presence of the NH signal *δ*_H_ 7.23 (d, *J* = 8.4 Hz). Chemical shift predictions were calculated using ChemNMR in ChemBioDraw 12.0, though improved methods exist as discussed in [39]. NMR chemical shift predictions provided strong support for **A**, as predictions compare well with **A** with *δ*_H_ 3.62 (H-1a), *δ*_H_ 3.99 (H-1b), *δ*_H_ 4.52 (H-3) and not **B** with *δ*_H_ 3.50 (H-1a), *δ*_H_ 3.25 (H-1b), *δ*_H_ 6.09 (H-3). 

We then turned to examine the geometry for the C-8 to C-9 olefin. Unfortunately, protons H-8 and H-9 overlapped and complicated the assignment due to secondary order effects. While attempts were made to shift protons H-8 and H-9 using solvent effects, the lack of solubility of **1** proved problematic, remarkably even in DMSO-*d*_6_ or pyridine-*d*_5_. Unfortunately of the over 20 solvent systems screened, **1** was only soluble in a mixtures of CDCl_3_/CD_3_OD (<5 mg/mL in 4:1 CD_2_Cl_2_/CD_3_OD), mixtures of CD_2_Cl_2_/CD_3_OD (<2 mg/mL in 5:1 CD_2_Cl_2_/CD_3_OD) and CD_3_OD (<0.5 mg/mL). Additionally, *J*-resolved 2D techniques, such as an E-COSY spectrum, also failed to provide additional support. Ultimately, the assignment of the C-8 to C-9 olefin was made using the original spectral dataset (Table 1), which supported the presence of trans-olefin at C-8 and C-9 with a 15.6 Hz coupling constant. Further support for this assignment came from evaluating the chemical shifts for C-7 (32.4) and C-10 (33.2). Based on the γ-effect [40], these carbons should be proximal to a trans-olefin (typically observed at 32–33 Hz), and not a cis-olefin (typically observed at 27–28 Hz). 

Fortunately, the analysis of the NMR spectrum from **3** in CDCl_3_ allowed comparison with previously reported data for *N*-((2*S*,3*R*,4*E*,8*E*)-1,3-dihydroxyoctadeca-4,8-dien-2-yl)-hexadecanamide [41] To our disappointment, the NMR data reported on *N*-((2*S*,3*R*,4*E*,8*E*)-1,3-dihydroxyoctadeca-4,8-dien-2-yl)-hexadecanamide contained significant reporting errors [41,42,43]. That aside, the consensus obtained by comparing multiple publications, as reported in Table 3, provided a match to ^1^H NMR and ^13^C NMR spectra of **3**. Finally, the optical rotation of **3** [α]_D_^20^ = −2.5 (*c* 0.5, CHCl_3_) was comparable to that reported for *N*-((2*S*,3*R*,4*E*,8*E*)-1,3-dihydroxyoctadeca-4,8-dien-2-yl)-hexadecanamide [43].

**Table 3:** Comparison of ^1^H NMR (500 MHz) and ^13^C NMR (125 MHz) data for ceramide **3** in CDCl_3_ with the data reported for *N*-((2*S*,3*R*,4*E*,8*E*)-1,3-dihydroxyoctadeca-4,8-dien-2-yl)-hexadecanamide ^a^ in CDCl_3_.

	Ceramide 3		*N*-((2*S*,3*R*,4*E*,8*E*)-1,3-dihydroxyoctadeca-4,8-dien-2-yl)-hexadecanamide ^a^	
	^1^H* δ*_H_ (mult, *J* in Hz)	^13^C* δ*_C_ (type)	^1^H* δ*_H_ (mult, *J* in Hz)	^13^C* δ*_C_ (type)
1a	3.67 (m) ^b^	62.5 (CH_2_)	3.70 (dd, 3.4, 11.0)	62.5 (CH_2_)
1b	3.92 (m)		3.95 (dd, 3.4, 11.0)	
2	3.89 (m) ^b^	54.8 (CH)	3.91 (m)	54.4 (CH)
3	4.29 (dd, 3.6, 6.6)	74.5 (CH)	4.32 (br t, 4.4)	74.7 (CH)
4	5.51 (dt, 15.5, 5.9)	129.3 (CH)	5.55 (dt, 15.4, 6.4)	129.2 (CH)
5	5.77 (dt, 16.0, 6.2)	133.6 (CH)	5.80 (dt, 15.4, 6.4)	133.5 (CH)
6	2.10 (dt, 6.6, 6.3)	32.1 ^c^ (CH_2_)	2.12 (m)	32.1 ^c^ (CH_2_)
7	2.05 (dt, 6.6, 6.3)	32.3 ^c^ (CH_2_)	2.08 (m)	32.3 ^c^ (CH_2_)
8	5.38 (dd, 17.2, 5.6)	129.1 (CH)	5.36 (dt, 15.2, 6.4)	127.0 ^f^ (128.9) (CH)
9	5.40 (dd, 17.2, 5.0)	131.5 (CH)	5.43 (dt, 15.2, 6.4)	131.4 (CH)
10	1.96 (td, 6.4, 6.8)	32.74 ^c^ (CH_2_)	1.97 (br dd, 2.2, 13.2) ^e^	32.6 ^c^ (CH_2_)
11–16	1.25 (m)	29.3–29.8, 32.1 ^d^	1.28 (m)	29.2–29.7, 31.9 ^d^
17	1.25 (m)	22.8 (CH_2_)	1.28 (m)	22.7 (CH_2_)
18	0.87 (t, 6.5)	14.2 (CH_3_)	0.88 (t, 6.8)	14.1 (CH_3_)
1′	-	174.3 (C=O)	-	174.0 (C=O)
2′	2.21 (t, 7.6)	37.0 (CH_2_)	2.23 (t, 7.4)	36.8 (CH_2_)
3′	1.62 (p, 7.2)	25.9 (CH_2_)	1.64 (br t, 7.4)	25.8 (CH_2_)
4′–14′	1.25 (m)	29.3–29.8, 32.1 ^d^	1.28 (m)	29.2–29.7, 31.9 ^d^
15′	1.25 (m)	22.8 (CH_2_)	1.28 (m)	22.7 (CH_2_)
16′	0.87 (t, 6.5)	14.2 (CH_3_)	0.88 (t, 6.8)	14.1 (CH_3_)
OH	2.99 (bs)	-	2.77 (bs)	-
N–H	6.33 (d, 7.4)	-	6.26 (d, 7.3)	-

^a^ Data presented was reported from a sample of (2*S*,2*R*,4*E*,8*E*)-*N*-hexadecanoyl-2-amino-4,8-octadecadiene-1,3-diol isolated from the coral *Dendronephthya gigantea* [41]; ^b^ Assignment of spin patterns for these peaks was questionable; ^c^ Peaks were too close to definitively assign via HSQC or HMBC analyses; ^d^ Two peaks were observed at 32.1 or 31.9, respectively, and all carbons were identified as methylenes (CH_2_); ^e^ Coupling constants were misinterpreted; ^f^ This value was likely reported incorrectly. The values presented in parentheses represent those reported by others [42,43].

Palyosulfonoceramide B (**2**) was obtained as white amorphous solid, mp 147 °C, and also displayed optical activity, [α]_D_^20^ = +6.7 (*c* 0.51, 4:1 CHCl_3_/MeOH). Compound **2** showed a similar IR spectrum as **1**. HR-ESI-MS analysis returned a molecular ion at *m/z* 657.5264 [M − H]^−^, two units higher than compound **1**, suggesting a molecular formula of C_37_H_74_N_2_O_5_S (calculated 657.5240). The ^1^H NMR spectrum of compound **2** was similar to **1** and contained one NH proton at *δ*_H_ 7.32 and *N*-methyl group at *δ*_H_ 2.51. Unlike **1**, only two vinyl protons were present at C-4 *δ*_H_ 5.26 (dd, *J* = 16.0 and 7.1 Hz) and C-5 *δ*_H_ 5.54 (m, H-5), indicating that C-8 to C-9 were saturated. This conclusion was in agreement with the two extra mass units observed by MS analysis.

While shifts in the ^1^H NMR data between **1** and **2** were observed due to difficulties in using exact solvent mixtures for the NMR analyses (note it was very difficult to use the same procedure to dissolve **1** and **2** once dry both required the first addition of CD_3_OD followed by addition of CDCl_3_), the coupling constants remained comparable (Table 1). As expected the ^13^C NMR spectrum of **2** contained a the carbonyl at *δ*_C_ 174.4 (C-1′), two olefinic carbon at *δ*_C_ 128.9 (C-4) and 134.3 (C-5), two oxygen-containing carbons at *δ*_C_ 71.3 (C-3) and 63.4 (C-1), two nitrogen-containing carbons at *δ*_C_ 53.9 (C-2) and 45.3 (C-2″) and a sulfur-containing carbon at *δ*_C_ 32.3 (C-2″). Assignment of the carbon spectrum was compatible with the two aliphatic side chains in **2** (Table 1). Ion-Daughter MS-MS analysis and two-dimensional NMR analyses (^1^H-^1^H COSY, ^1^H-^13^C HSQC, ^1^H-^13^C HMBC and ^1^H-^15^N HSQC) definitively agreed with the structure suggested for **2**. 

Further evidence the assignment of **1** and **2** came from the evaluation of ceramide **4**. Like **3**, the NMR data collected from **4** in CDCl_3_ matched that published for *N*-((2*S*,3*R*,4*E*)-1,3-dihydroxyoctadeca-4,8-dien-2-yl)-hexadecanamide (Table 4) [43]. In addition, HR-ESI-MS analysis provided molecular ion [M − H]^−^ for ceramide **4 **at *m/z* 536.5039 compatible with a molecular formula of C_34_H_67_NO_3_ (calculated [M − H]^−^ at *m/z* 536.5043). This along with the optical rotation of **4** [α]_D_^20^ = −5.2 (*c* 0.11, CHCl_3_) was comparable the reported value [43] indicating that **4** was *N*-((2*S*,3*R*,4*E*,8*E*)-1,3-dihydroxyoctadeca-4,8-dien-2-yl)-hexadecanamide. Comparison of the data obtained between **1**, **2** and co-isolated ceramides **3** and **4**, along with the published data on **3** and **4** provides a definitive argument for their assignment as shown in Figure 2. Compounds **1** and **2** represent new materials bearing a unique sulfonylated group, a functionality that has to date only been reported as a synthetic intermediate [37,38]. 

**Table 4:** Comparison of ^1^H NMR (500 MHz) and ^13^C NMR (125 MHz) data for ceramide **4 **in CDCl_3_ with the data reported for *N*-((2*S*,3*R*,4*E*)-1,3-dihydroxyoctadeca-4,8-dien-2-yl)-hexadecanamide ^a^ in CDCl_3_.

	Ceramide 4		*N*-((2*S*,3*R*,4*E*)-1,3-dihydroxyoctadeca-4,8-dien-2-yl)-hexadecanamide ^a^	
	^1^H *δ*_H_ (mult, *J* in Hz)	^13^C *δ*_C_ (type)	^1^H *δ*_H_ (mult, *J* in Hz)	^13^C *δ*_C_ (type)
1a	3.67 (dd, 3.4, 11.3)	62.7 (CH_2_)	3.70 (dd, 3.2, 11.2)	62.5 (CH_2_)
1b	3.96 (dd, 3.3, 11.3)		3.95 (dd, 3.9, 11.2)	
2	3.92 (qd, 3.7, 7.5)	54.6 (CH)	3.90 (m)	54.5 (CH)
3	4.33 (dd, 4.4, 6.0)	74.9 (CH)	4.31 (dd, 4.4, 6.8)	74.7 (CH)
4	5.53 (tdd, 1.4, 6.4, 15.5)	128.9 (CH)	5.53 (td, 6.8, 15.5)	128.9 (CH)
5	5.79 (dtd, 1.2, 6.8, 15.2)	134.5 (CH)	5.78 (td, 6.6, 15.2)	134.3 (CH)
6	2.06 (m)	32.1 (CH_2_)	2.05 (m)	32.3 (CH_2_)
7–17	1.25 (m)	22.9–32.1 (CH_2_)	1.25 (m)	22.7–31.9 (CH_2_)
18	0.88 (t, 6.9)	14.3 (CH_3_)	0.87 (t, 6.6)	14.1 (CH_3_)
1′	-	174.0 (C=O)	-	173.9 (C=O)
2′	2.23 (dd, 7.1, 8.2)	37.0 (CH_2_)	2.22 (t, 7.8)	36.8 (CH_2_)
3′	1.62 (m)	25.9 ^b^ (CH_2_)	1.63 (m)	25.7 ^b^ (CH_2_)
4′–15′	1.25 (m)	22.7–32.1 (CH_2_)	1.25 (m)	22.7–31.9 (CH_2_)
16′	0.88 (t, 6.9)	14.3 (CH_3_)	0.87 (t, 6.6)	14.1 (CH_3_)
OH	2.59 (bs)	-	2.68 (bs)	-
N–H	6.22 (d, 7.6)	-	6.24 (d, 7.3)	-

^a^ Data presented was collected from sample of (2*S*,2*R*,4*E*)-*N*-hexadecanoyl-2-amino-4,8-octadecadiene-1,3-diol isolated from the gorgonian *Acabaria undulata* [43]; ^b^ Tenative assignment.

Ceramides have a significant potential for biological applications, being a suitable source for new biological probes and therapeutic leads [44], as recently established through the preparation of ceramide libraries [45,46]. A wide variety of biological effects have been reported for cerebrosides, glycosylated-ceramides, including cytotoxic, antitumor, antiviral, antifungal, immunostimulating and immunosuppressive [44]. Here, the antiproliferative potential of ceramides **1**–**4** was investigated against the HCT-116 colon adenocarcinoma cell line, however they remained inactive at the highest tested concentration at 50 μg/mL. In multiple related studies, the sugar moiety has been determined as critical to cyctotoxic activity [44]. For instance, Zeng demonstrated that the presence of a glucose unit in cerebrosides isolated from a plant *Livistona chinensis* increase cytotoxicity by 10–20 fold [47]. As **1** and **2** are ceramides, and not cerebrosides (lack glycosylation), it is not surprising they lack a cytotoxic response. Chemical modifications of these materials may be an interesting approach to expand their biological properties. Moreover, this structure class offers additional input to the design of ceramide-derived libraries [45,46]. In addition, compounds **1** and **2** may play a biological role that is not related to cell death. Efforts are now underway to further explore their biological function.

## 3. Experimental Section

### 3.1. General Experimental Procedures

Melting points were obtained on a digital MQAPF-302 melting point apparatus (Microquimica). Optical rotations were measured on a 341 digital polarimeter (Perkin-Elmer, Waltham, MA, USA). Fourier transform infrared (FT-IR) spectra were acquired on a Spectrum 1000 spectrometer (Perkin-Elmer, Waltham, MA, USA). The high-resolution electrospray ionization mass spectra (HR-ESI-MS) were acquired using a LCMS-IT-TOF spectrometer (Shimadzu, Shimane, Japan). ^1^H NMR (500 MHz) and ^13^C NMR (125 MHz) spectra were collected on a DRX-500 spectrometer (Bruker, Rheinstetten, Germany). HPLC analysis was carried out using a ultrafast liquid chromatography (UFLC) system (Shimadzu) equipped with an SPD-M20A diode array UV-Vis detector and a 5 μm (4.6 × 250 mm) C-18 column (Phenomenex, Torrance, CA, USA). Chromatography columns were carried out on: silica gel 60 with 70–230 mesh (Vetec, Rio de Janeiro, Brazil); silica gel 60 with 230–400 mesh (Merck, Darmstadt, Germany); Sephadex LH-20; or Strata C18-E, 20 g/60 mL, 55 μm, 70 Å cartridges (Phenomenex, Torrance, CA, USA). Thin-Layer chromatography (TLC) was performed on silica gel aluminum sheets coated with 0.2 mm silica gel 60 F_254_ (Merck, Darmstadt, Germany). TLC plates were visualized sprayed with vanillin/HClO_4_/EtOH solution and visualized by heating at ~100 °C.

### 3.2. Marine Organisms

The zoanthids *Palythoa caribaeorum *and *Protopalythoa variabilis *were collected at the low tide from Paracuru beach (3°24′0.22″S and 39°0′48.60″W), a site that is ~93 km from Fortaleza, Brazil. Specimen identification was conducted by Antonio Carlos Marques at the Universidade de São Paulo. Samples of each specimen, voucher numbers 000976 and 000975, respectively, were deposited in the Universidade de São Paulo Zoology Museum (MUZUSP-USP). 

### 3.3. Extraction and Isolation

*P. caribaeorum* (4.7 kg) was cut in small pieces and washed with distilled water. The pieces were soaked in *n*-hexane (6 L) at rt for 24 h. The *n*-hexane layer was collected and concentrated via rotary evaporation with the bath temperature ≤40 °C to afford 21 g of a crude extract. The resulting extract was then fractionated on a silica gel column (108 g, 7 cm i.d.) with sequential elution with *n*-hexane, 4:1 *n*-hexane/EtOAc, 3:2 *n*-hexane/EtOAc, 2:3 *n*-hexane/EtOAc, 1:4 *n*-hexane/EtOAc, EtOAc and finally MeOH. The MeOH fraction (230.2 mg), a yellowish amorphous powder, was subjected to column chromatography over silica gel (15 g, 3 cm ID) using an elution of 9:1 CH_2_Cl_2_/MeOH, 4:1 CH_2_Cl_2_/MeOH, 7:3 CH_2_Cl_2_/MeOH, 3:2 CH_2_Cl_2_/MeOH, 1:1 CH_2_Cl_2_/MeOH to MeOH. The 1:1 CH_2_Cl_2_/MeOH fraction (137.2 mg), a white amorphous powder, was further purified by semi-preparative reverse HPLC with MeOH as the mobile phase at a flow rate of 4.7 mL·min^−1^ for 20 min using a 251–400 nm monitor to afford **1 **(35.6 mg, *t_R_* 8.0 min) and **2 **(28.3 mg, *t_R_* 9.0 min). Compounds **3** and **4** were obtained from the 1:4 *n*-hexane/EtOAc (1.1 g) fraction by purification via silica gel column chromatography (22 g, 3.5 cm i.d.) eluting with 1:1 *n*-hexane/EtOAc, 2:3 *n*-hexane/EtOAc, 3:7 *n*-hexane/EtOAc, 1:4 *n*-hexane/EtOAc, 1:9 *n*-hexane/EtOAc, EtOAc and finally MeOH. The sub-fractions 1:1 *n*-hexane/EtOAc and 2:3 *n*-hexane/EtOAc were joined and dried to afford 146.7 mg of an white amorphous powder. Semi-Preparative reverse phase HPLC with MeOH as the mobile phase at a flow rate of 4.7 mL·min^−1^ for 20 min using a 251-400 nm monitor to afforded **3 **(37.1 mg, *t_R_* 8.2 min) and **4 **(17.8 mg, *t_R_* 9.1 min). Application of these procedures to *P. variabilis *extracts (3.4 kg) yielded 34.6 mg of **1**, 27.7 mg of **2**, 31.4 mg **3** and 15.8 mg **4**.

### 3.4. Methanolysis and GC-MS Analysis

Aliquots of compounds **1**–**4** (5 mg each) were heated individually with 5% HCl in MeOH (5 mL) at 70 °C for 2 h. The reaction mixture was then extracted with *n*-hexane, and the extract was concentrated under vacuum to yield the corresponding methyl ester. A sample (1 mg) of each was analyzed by GC-MS. The GC-MS analysis was carried out on a Shimadzu GC-17A/QP-5050 using a non-polar DB-1 fused silica capillary column (30 m × 0.25 mm i.d., 0.25 μm film thickness); carrier gas helium, flow rate 1 mL·min^−1^ and with split mode (ratio 1:48). The injector temperature and detector temperature were 250 °C and 280 °C, respectively. The column temperature was programmed from 40 °C to 180 °C at 4 °C min^−1^ and then 180 °C to 250 °C at 20 °C min^−1^ and held isothermal for 7 min. 

### 3.5. Cytotoxicity Assays

Palyosulfonoceramides A (**1**) and B (**2**) and ceramides *N*-((2*S*,3*R*,4*E*,8*E*)-1,3-dihydroxyoctadeca-4,8-dien-2-yl)-hexadecanamide (**3**) and *N*-((2*S*,3*R*,4*E*)-1,3-dihydroxyoctadeca-4-en-2-yl)-hexadecanamide (**4**) were evaluated for their cytotoxic effect against a human colon adenocarcinoma cell line (HCT-116) using the 3-(4,5-dimethyl-2-thiazolyl)-2,5-diphenyl-2*H*-tetrazolium bromide (MTT) assay [48]. Cells were plated into 96-well plates (1 × 10^5^ cells/mL) and cultured for 24 h prior to addition of tested substances. Compounds were screened by addition of a single dose ranging from 0.01 to 50 μg/mL followed by incubation for at 37 °C for 72 h. Control groups received 0.1% of vehicle used to diluted the tested substances (CHCl_3_/MeOH). Three hours before the end of the incubation, 150 μL of a stock solution (0.5 mg/mL) of MTT (Sigma-Aldrich Co., Saint Louis, MO, USA) was added to each well. Absorbance was measured using a DTX 880 Multimode multiplate reader (Beckman Coulter Inc., Fullerton, CA, USA). Data was repeated in triplicate for each compound and control screened.

## 4. Conclusions

We report the isolation of two novel sulfonylated ceramides, palyosulfonoceramide A (**1**) and palyosulfonoceramide B (**2**), from extracts of *P. caribaeorum* and *P. variabilis* collected from the Northeastern coast of Brazil. The structures of **1** and **2** were determined by a combination of NMR and HRMS methods along with comparison co-isolated ceramides **3** and **4** with literature precedent. Compounds **1** and **2** represent a new class of sulfur-containing lipids and present new structural motifs for further biological inquiry. 

## Supplementary Files

Supplementary File 1 Supplementary Information (PDF, 2609 KB)
