# Supplementary material for: Palyosulfonoceramides A and B: Unique Sulfonylated Ceramides from the Brazilian Zoanthids Palythoa caribaeorum and Protopalyhtoa variabilis
Source: Mar Drugs. 2012 Dec 14;10(12):2846–60. doi: 10.3390/md10122846 (PMC3528130; doi:10.3390/md10122846)

Supplementary Information

Figure S1. HR-ESI-MS spectrum of **1**. 2

Figure S2. HR-ESI-MS-MS spectrum of **1**. 2

Figure S3. HPLC chromatogram of **1**. 2

Figure S4. Infrared spectrum of **1**. 3

Figure S5. ^{1}H-NMR spectrum (500 MHz, 4:1 $CDCl_3$/CD_3OD) of **1**. 3

Figure S6. ^{13}C-NMR spectrum (125 MHz, 4:1 $CDCl_3$/CD_3OD) of **1**. 4

Figure S7. ^{1}H–^{1}H-COSY spectrum (500 × 500 MHz, 4:1 $CDCl_3$/CD_3OD) of **1**. 5

Figure S8. ^{1}H–^{13}C-HSQC spectrum (500 × 125 MHz, 4:1 $CDCl_3$/CD_3OD) of **1**. 6

Figure S9. ^{1}H–^{13}C-HMBC spectrum (500 × 125 MHz, 4:1 $CDCl_3$/CD_3OD) of **1**. 7

Figure S10. ^{1}H–^{15}N-HMBC spectrum (500 × 50 MHz, 4:1 $CDCl_3$/CD_3OD) of **1**. 8

Figure S11. HR-ESI-MS spectrum of **2**. 8

Figure S12. HR-ESI-MS-MS spectrum of **2**. 9

Figure S13. ^{1}H-NMR spectrum (500 MHz, 4:1 $CDCl_3$/CD_3OD) of **2**. 9

Figure S14. ^{13}C-NMR spectrum (125 MHz, 4:1 $CDCl_3$/CD_3OD) of **2**. 10

Figure S15. HSQC spectrum (500 × 125 MHz, 4:1 $CDCl_3$/CD_3OD) of **2**. 11

Figure S16. ^{1}H–^{13}C-HMBC spectrum (500 × 125 MHz, 4:1 $CDCl_3$/CD_3OD) of **2**. 12

Figure S17. ^{1}H–^{15}N-HMBC spectrum (500 × 50 MHz, 4:1 $CDCl_3$/CD_3OD of **2**. 13

Figure S18. HR-ESI-MS spectrum of **3**. 13

Figure S19. ^{1}H-NMR spectrum (500 MHz, pyridine-d_5) of **3**. 14

Figure S20. ^{13}C-NMR spectrum (125 MHz, pyridine-d_5) of **3**. 14

Figure S21. ^{1}H–^{1}H-COSY spectrum (500 × 500 MHz, pyridine-d_5) of **3**. 15

Figure S22. ^{1}H–^{13}C-HSQC spectrum (500 × 125 MHz, pyridine-d_5) of **3**. 15

Figure S23. ^{1}H–^{13}C-HMBC spectrum (500 × 125 MHz, pyridine-d_5) of **3**. 16

Figure S24. HR-ESI-MS spectrum of **4**. 16

Figure S25. ^{1}H-NMR spectrum (500 MHz, pyridine-d_5) of **4**. 17

Figure S26. ^{13}C-NMR spectrum (125 MHz, pyridine-d_5) of **4**. 18

Figure S27. ^{1}H–^{1}H-COSY spectrum (500 × 500 MHz, pyridine-d_5) of **4**. 19

Figure S28. ^{1}H–^{13}C-HSQC spectrum (500 × 125 MHz, pyridine-d_5) of **4**. 20

Figure S29. ^{1}H–^{13}C-HMBC spectrum (500 × 125 MHz, pyridine-d_5) of **4**. 21

Figure S30. ^{1}H-NMR spectrum (500 MHz, $CDCl_3$) of **3**. 22

Figure S31. ^{13}C-NMR spectrum (125 MHz, $CDCl_3$) of **3**. 23

Figure S32. ^{1}H–^{13}C-HSQC spectrum (500 × 125 MHz, $CDCl_3$) of **3**. 23

Figure S33. ^{1}H-NMR spectrum (500 MHz, $CDCl_3$) of **4**. 24

Figure S34. ^{13}C-NMR spectrum (125 MHz, $CDCl_3$) of **4**. 25

Figure S1. HR-ESI-MS spectrum of **1**.

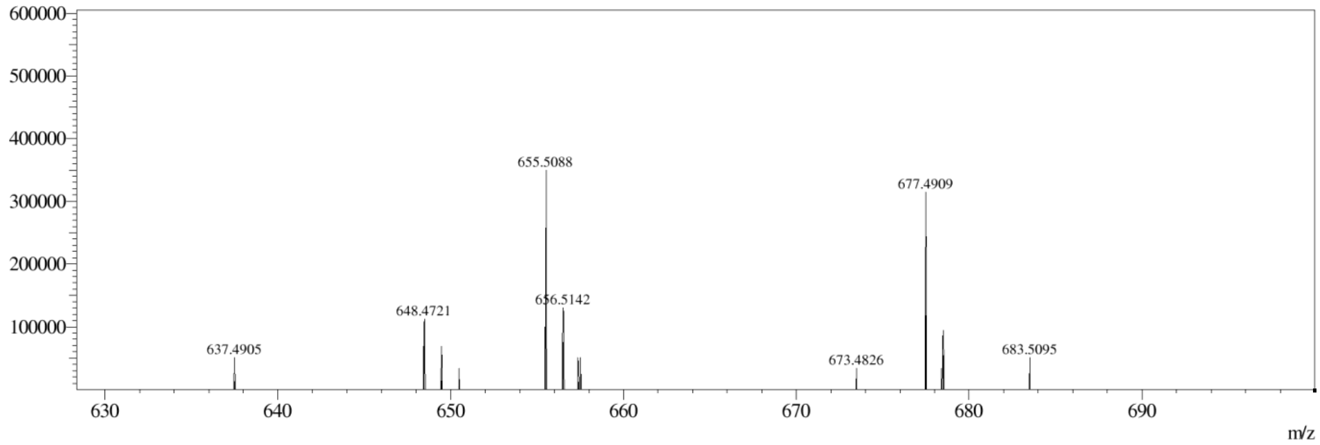

Figure S2. HR-ESI-MS-MS spectrum of **1**.

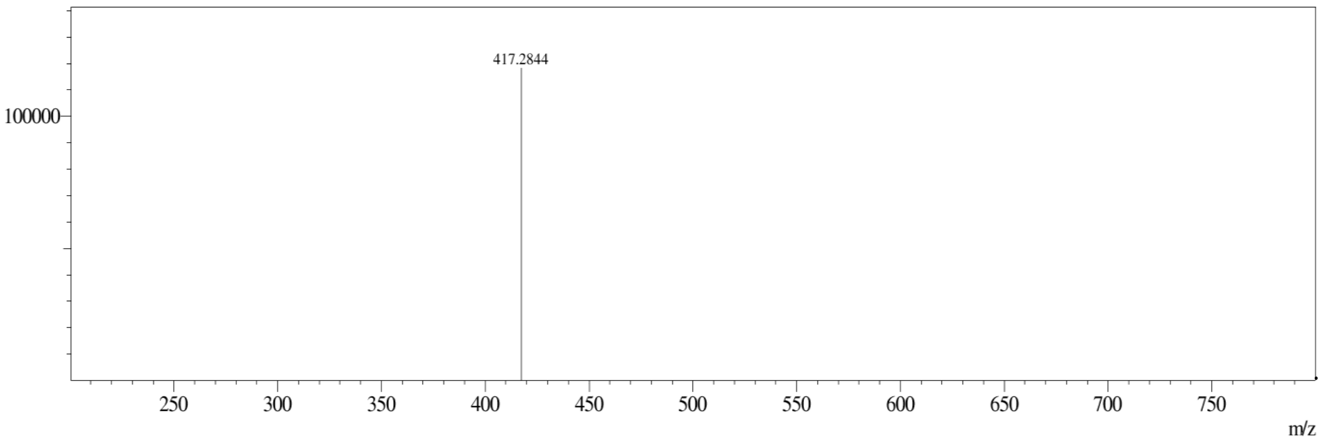

Figure S3. HPLC chromatogram of **1**.

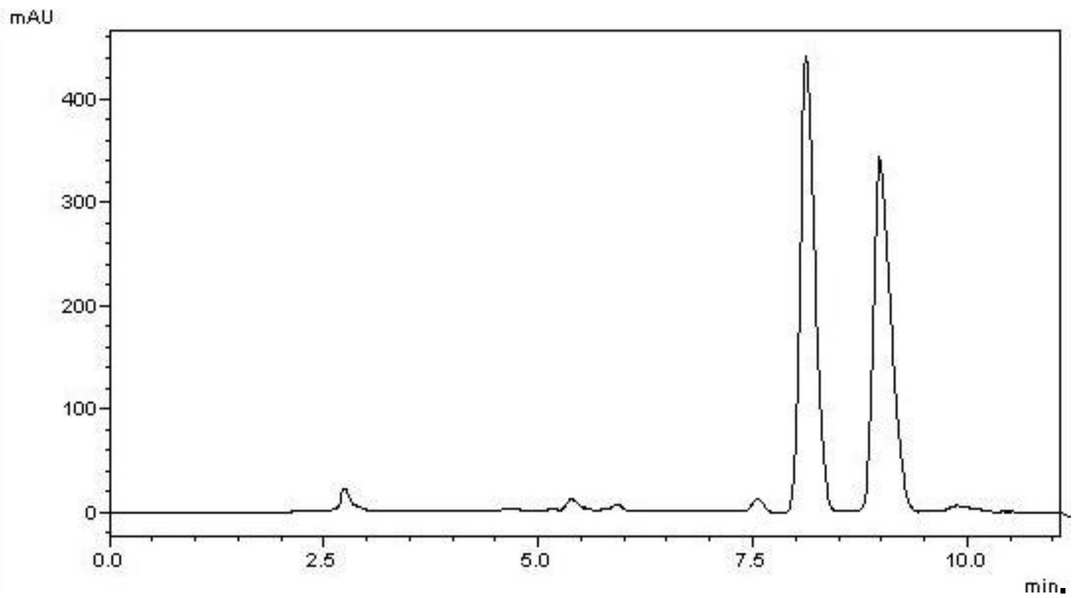

Figure S4. Infrared spectrum of **1**.

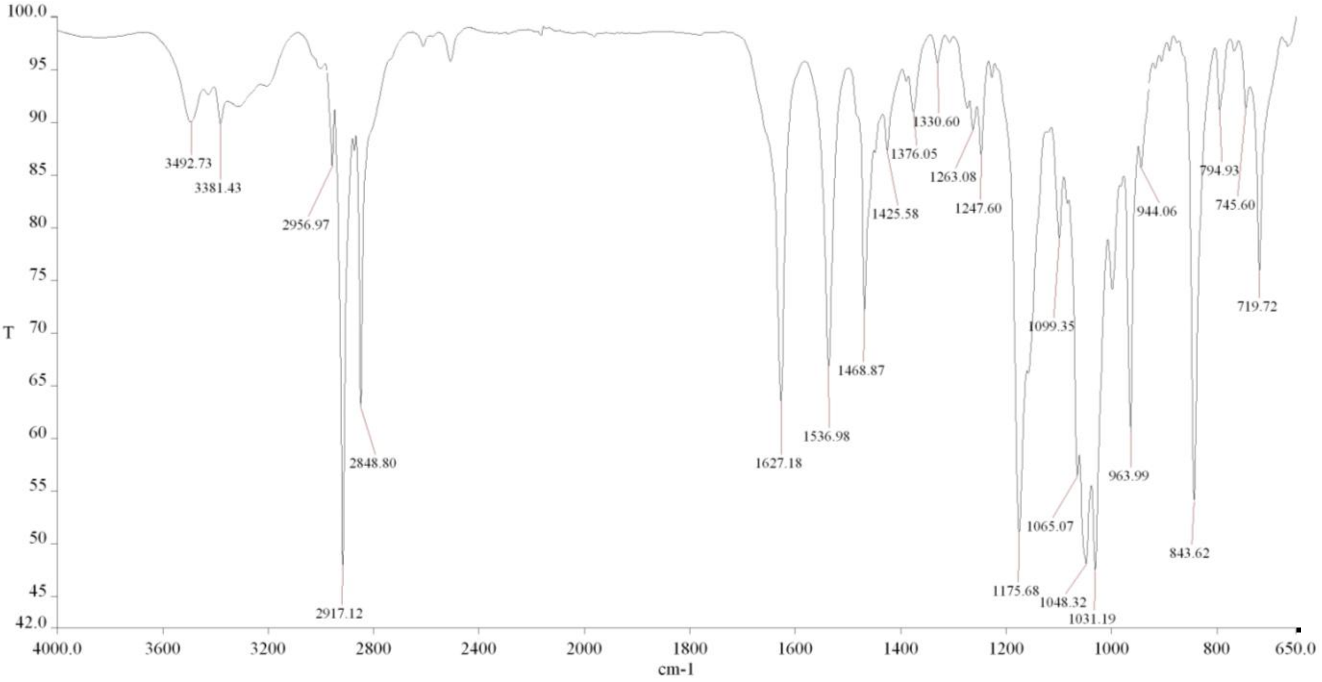

Figure S5. ^{1}H-NMR spectrum (500 MHz, 4:1 $CDCl_3/CD_3OD$) of **1**. (bottom) Expansion of the olefin region depicting assignments of protons H4, H5, H8 and H9.

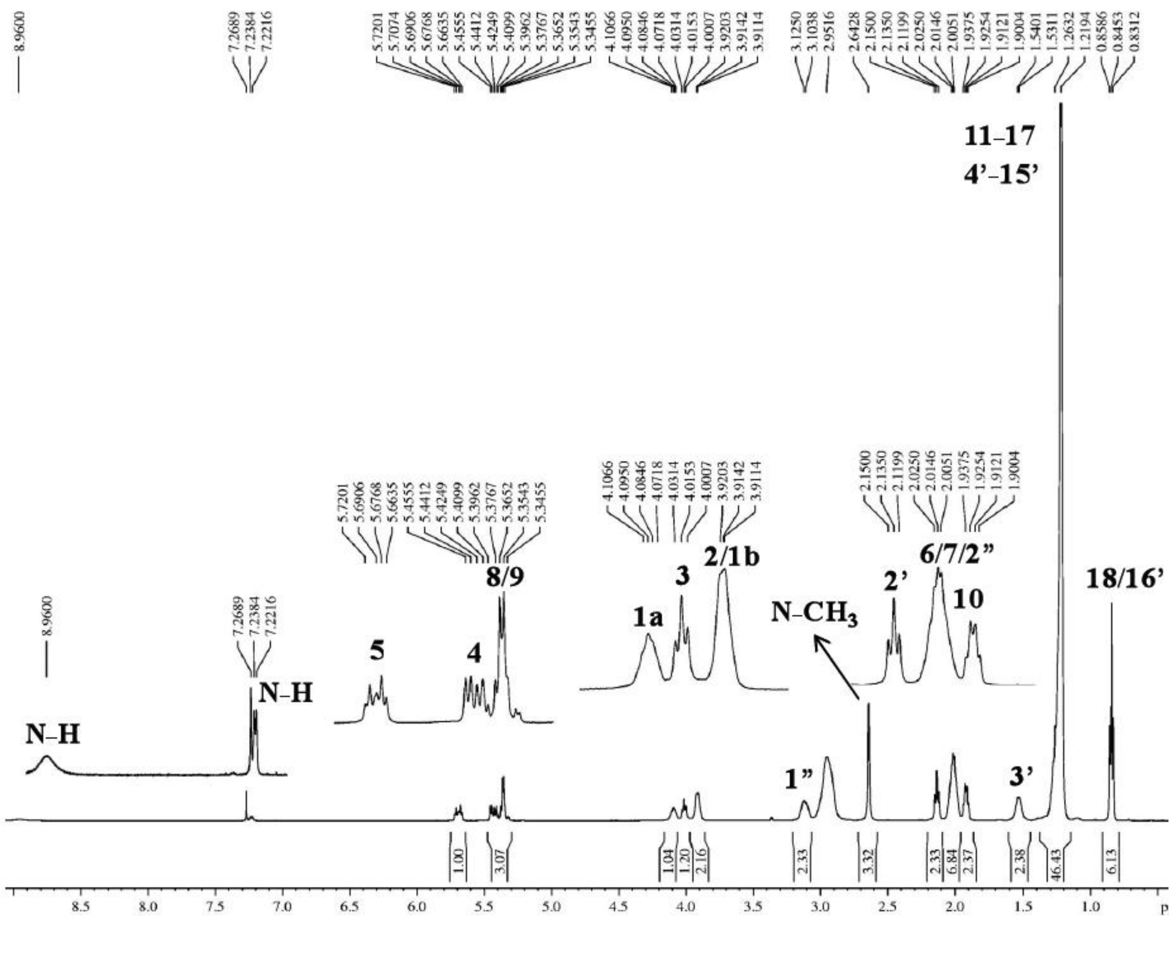

Figure S5. *Cont.*

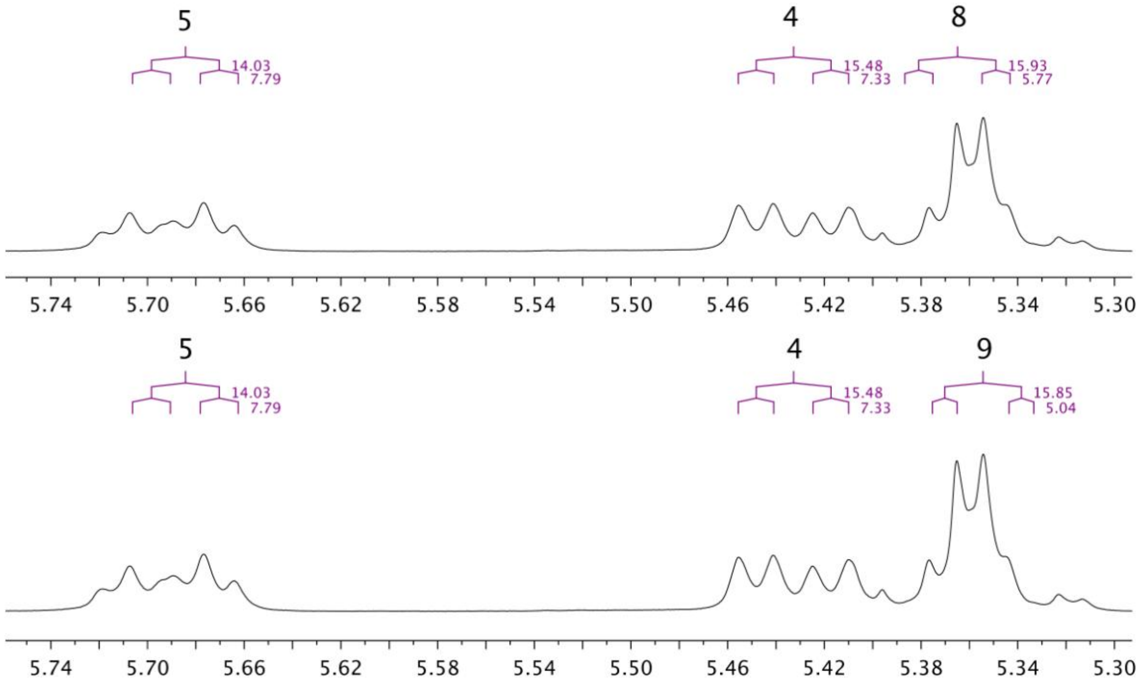

Figure S6. ^{13}C-NMR spectrum (125 MHz, 4:1 $CDCl_3/CD_3OD$) of **1**.

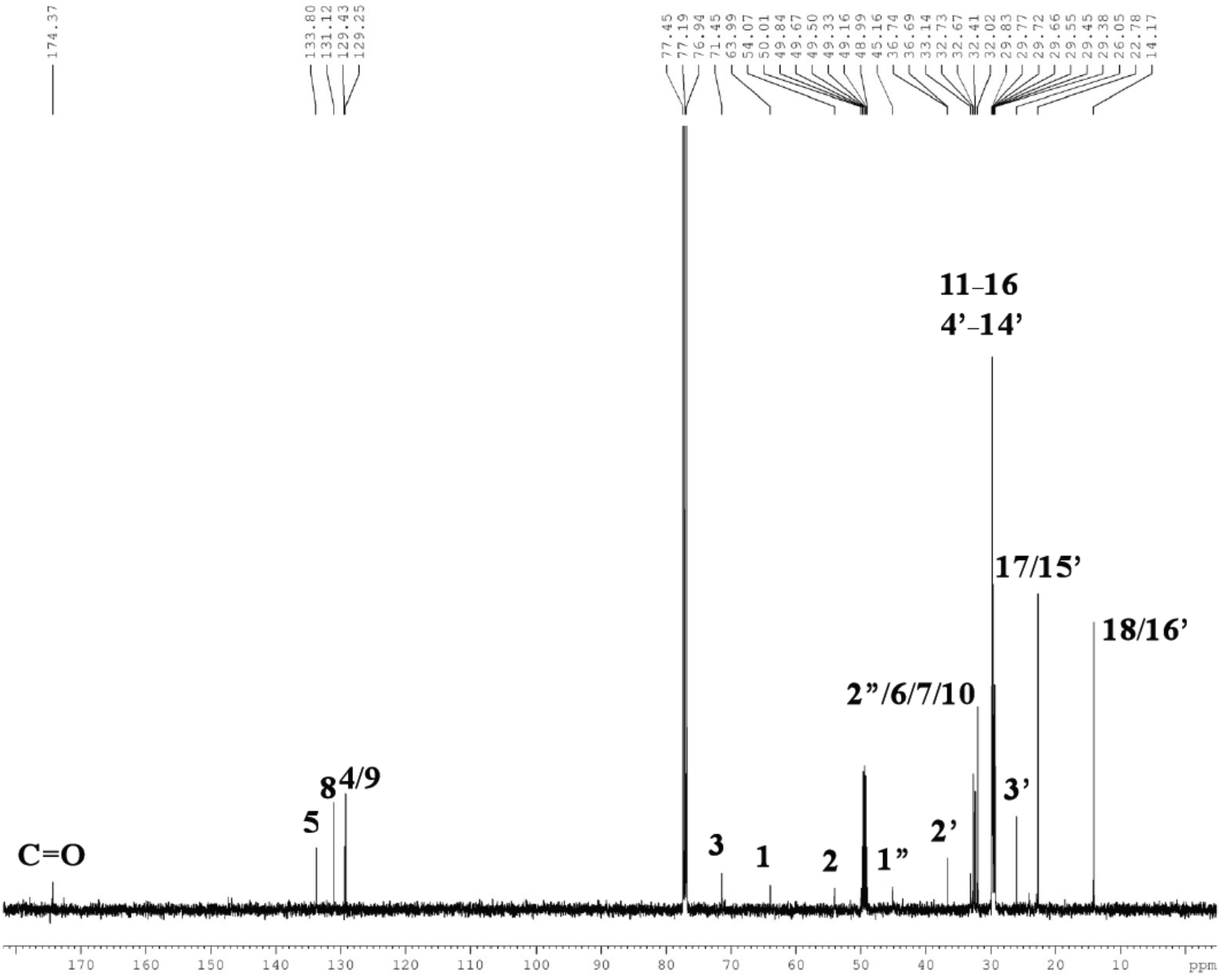

Figure S7. ^{1}H–^{1}H-COSY spectrum (500 × 500 MHz, 4:1 $CDCl_3$/CD_3OD) of **1**.

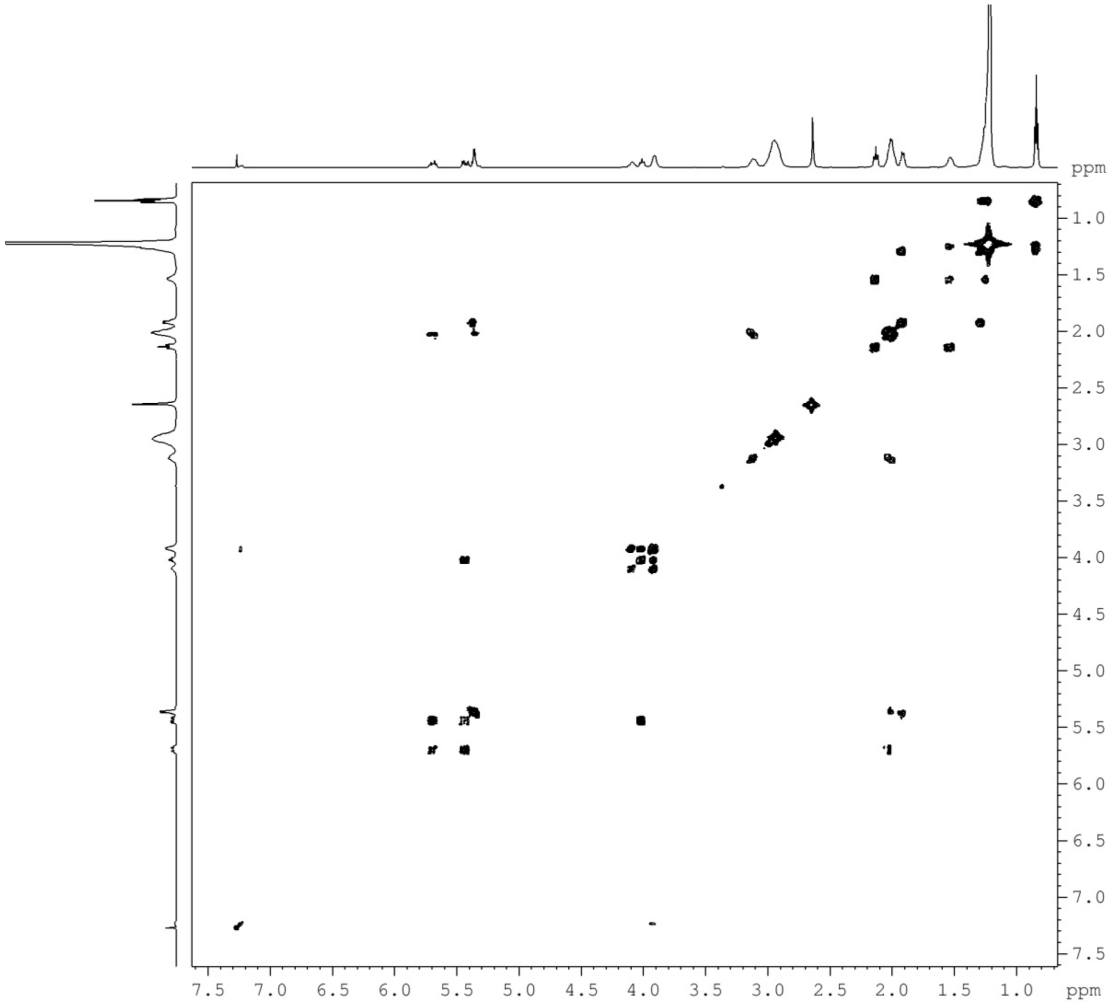

Figure S8. ^{1}H–^{13}C-HSQC spectrum (500 × 125 MHz, 4:1 $CDCl_3/CD_3OD$) of **1**.

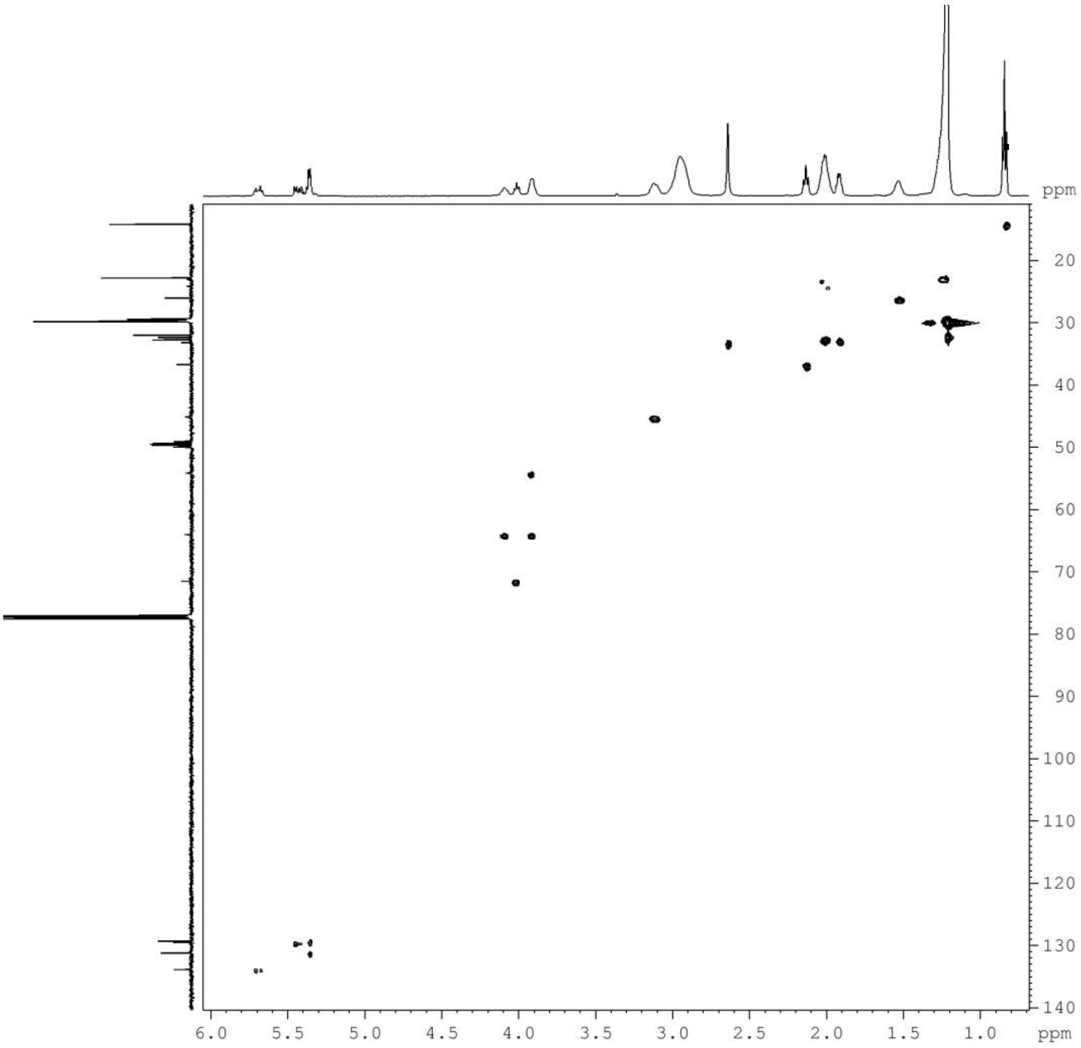

Figure S9. ^{1}H–^{13}C-HMBC spectrum (500 × 125 MHz, 4:1 $CDCl_3/CD_3OD$) of **1**.

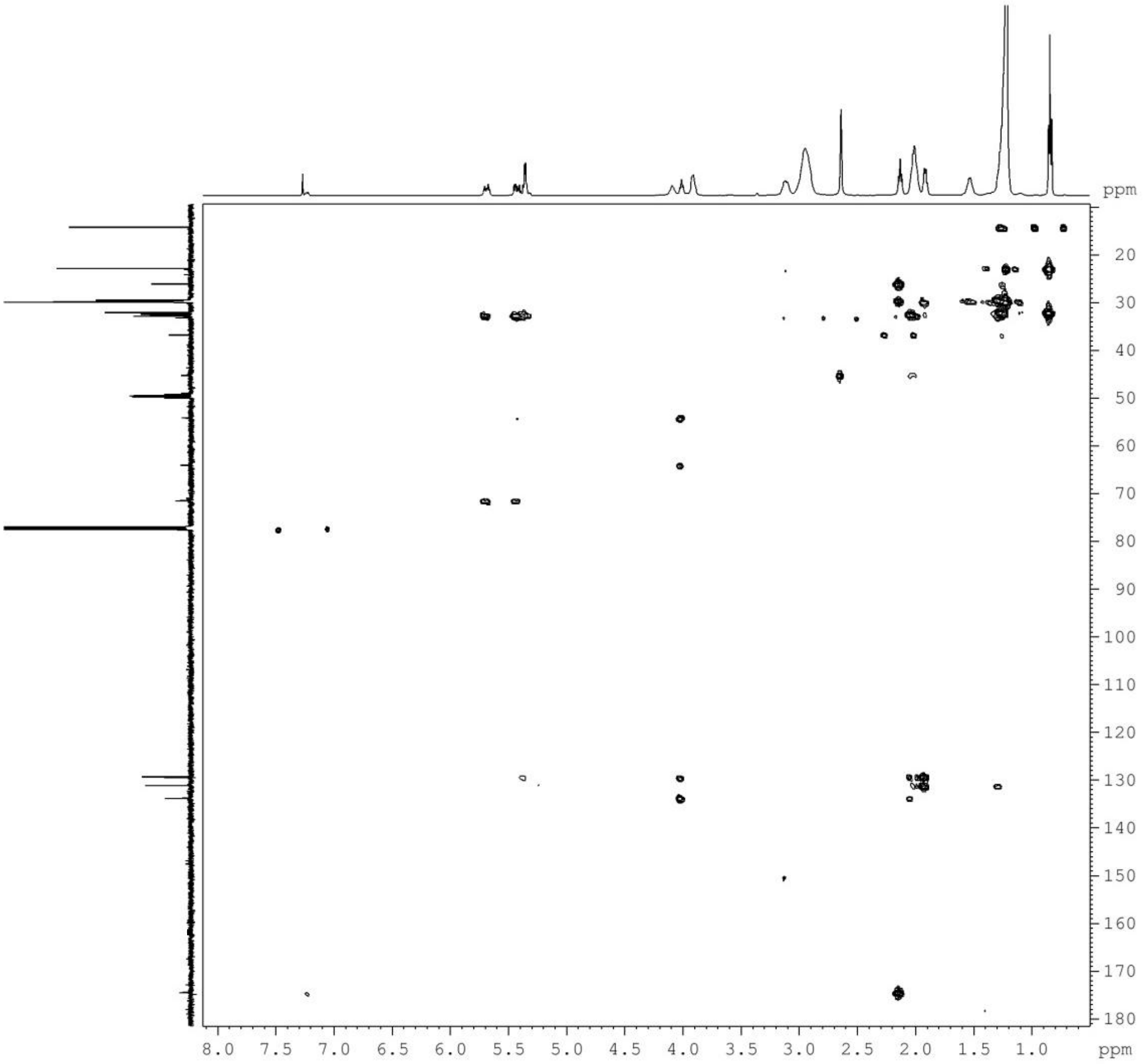

Figure S10. ^{1}H–^{15}N-HMBC spectrum (500 × 50 MHz, 4:1 $CDCl_3$/CD_3OD) of **1**.

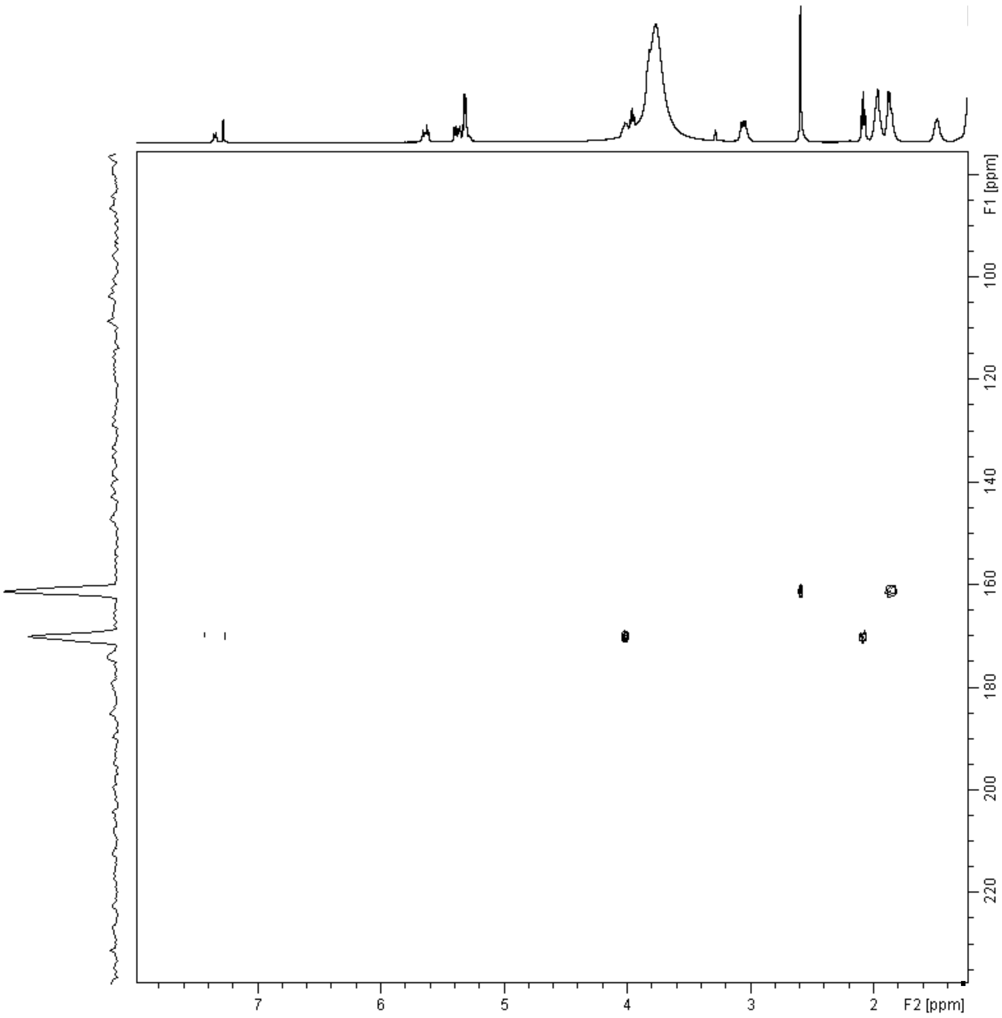

Figure S11. HR-ESI-MS spectrum of **2**.

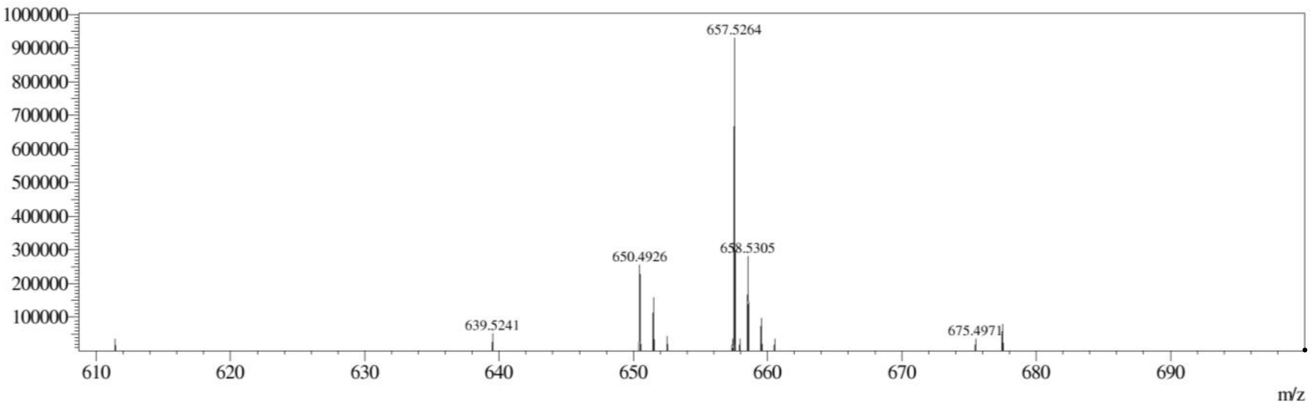

Figure S12. HR-ESI-MS-MS spectrum of **2**.

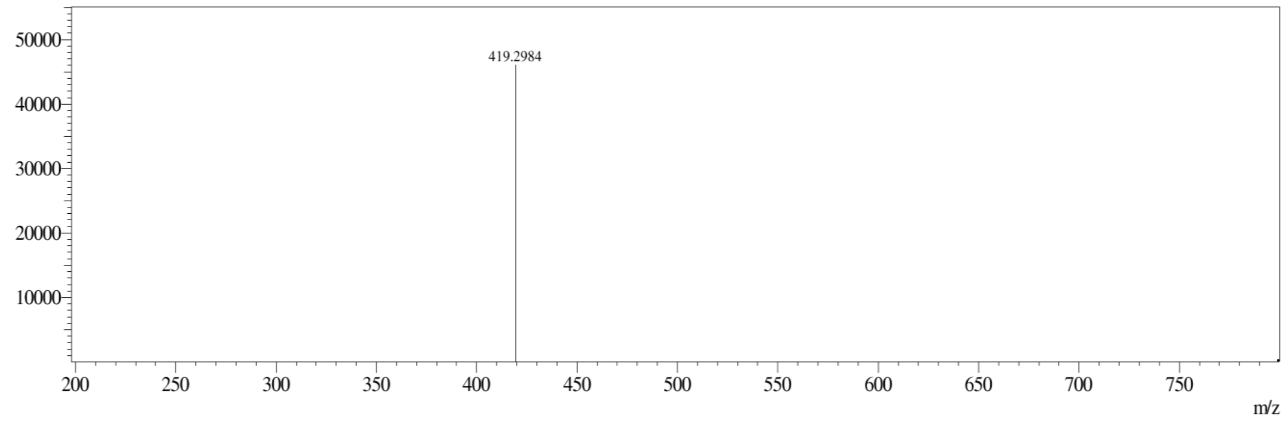

Figure S13. ^{1}H-NMR spectrum (500 MHz, 4:1 $CDCl_3/CD_3OD$) of **2**.

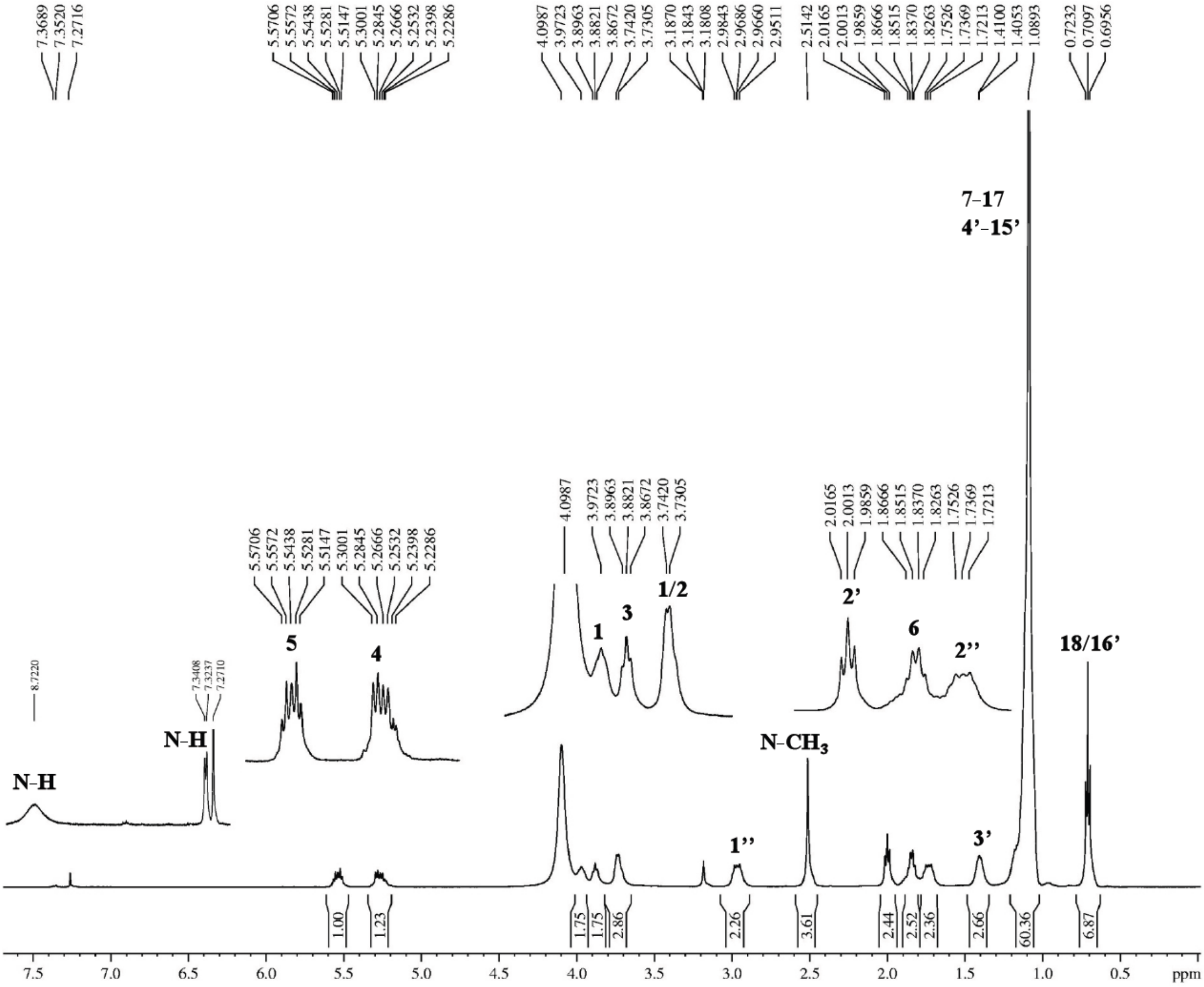

Figure S14. ^{13}C–NMR spectrum (125 MHz, 4:1 $CDCl_3/CD_3OD$) of **2**.

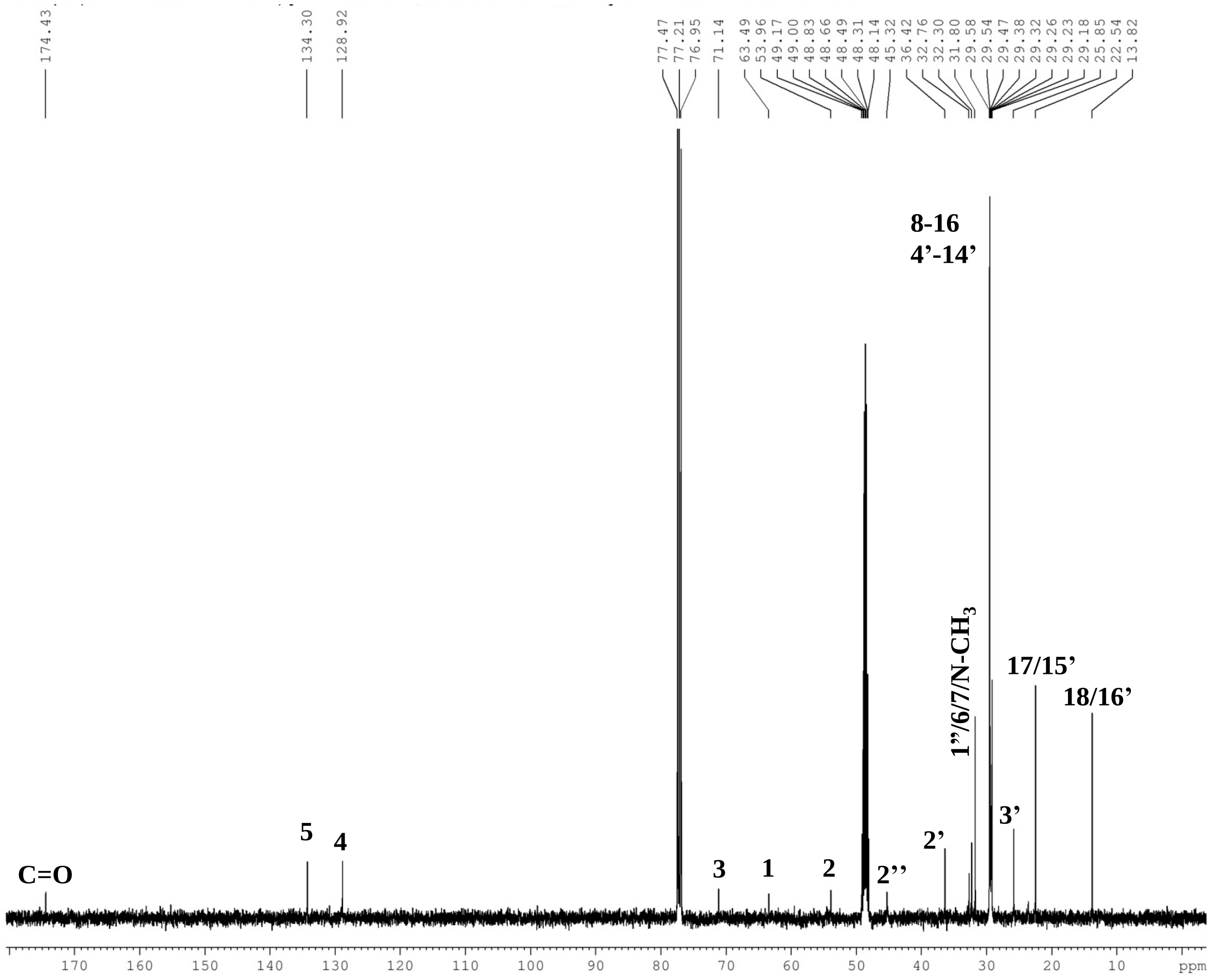

Figure S15. ^{1}H–^{13}C-HSQC spectrum (500 × 125 MHz, 4:1 $CDCl_3/CD_3OD$) of **2**.

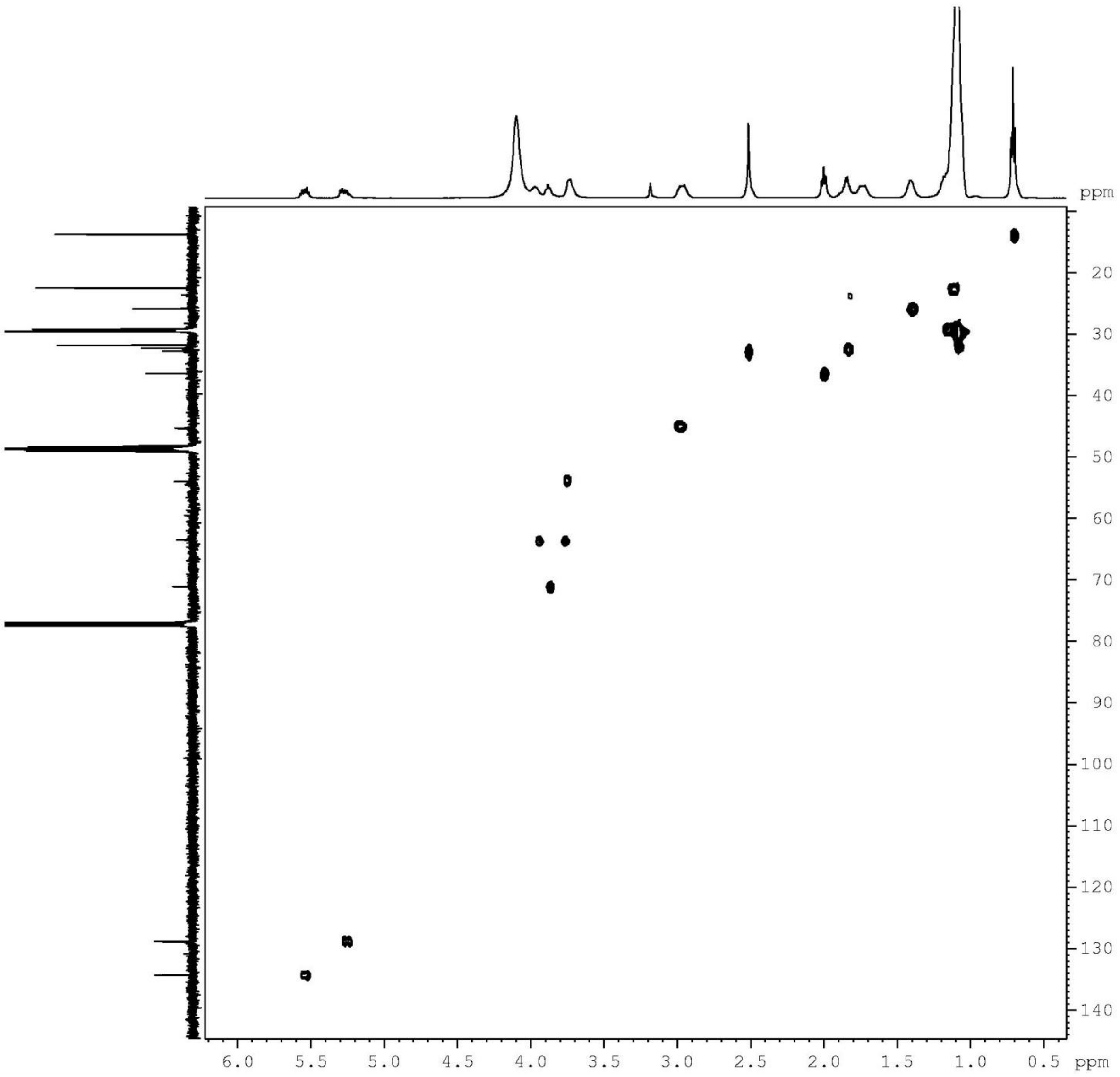

Figure S16. ^{1}H–^{13}C-HMBC spectrum (500 × 125 MHz, 4:1 $CDCl_3/CD_3OD$) of **2**.

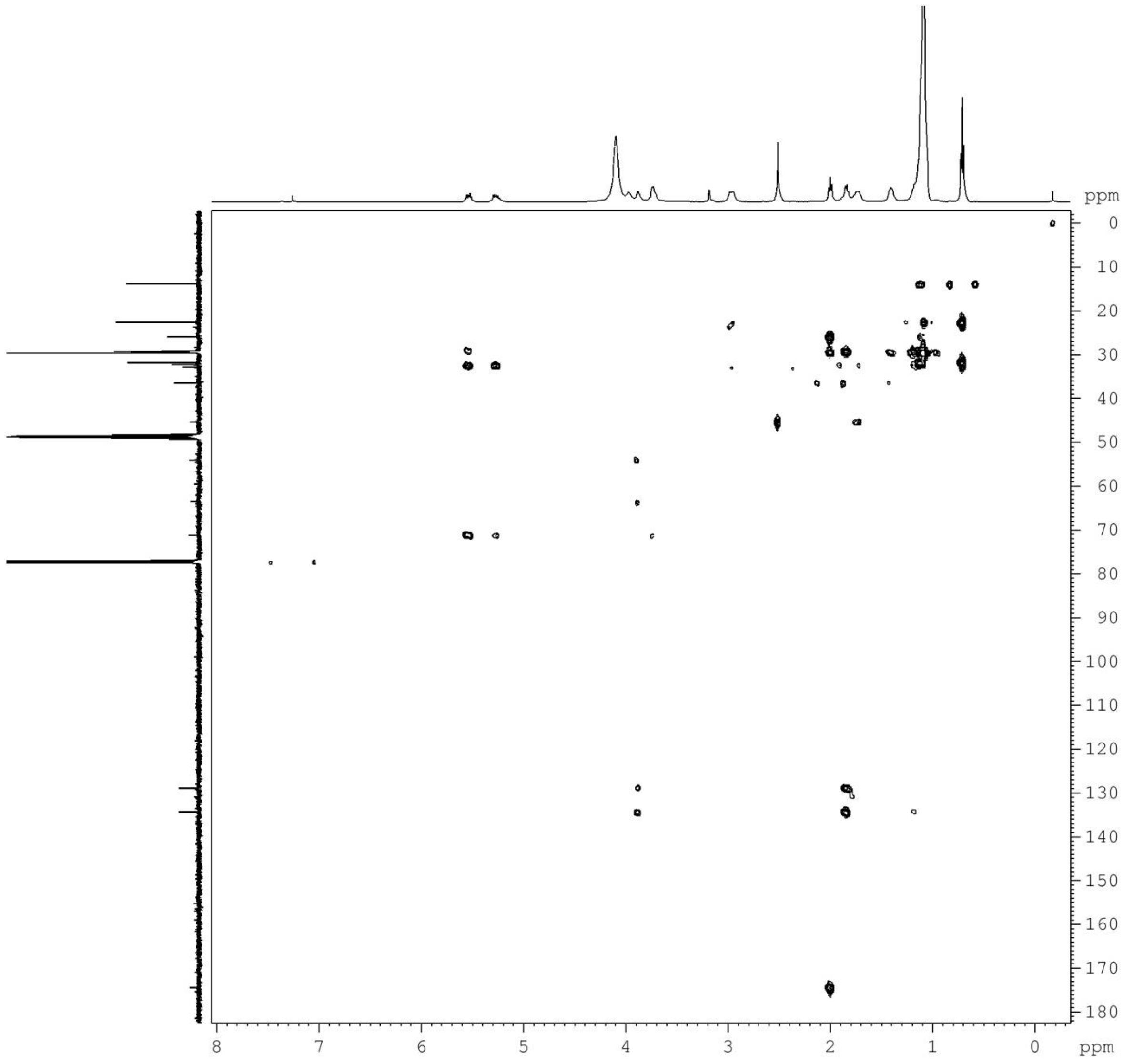

Figure S17. ^{1}H–^{15}N-HMBC spectrum (500 × 50 MHz, 4:1 $CDCl_3/CD_3OD$) of **2**.

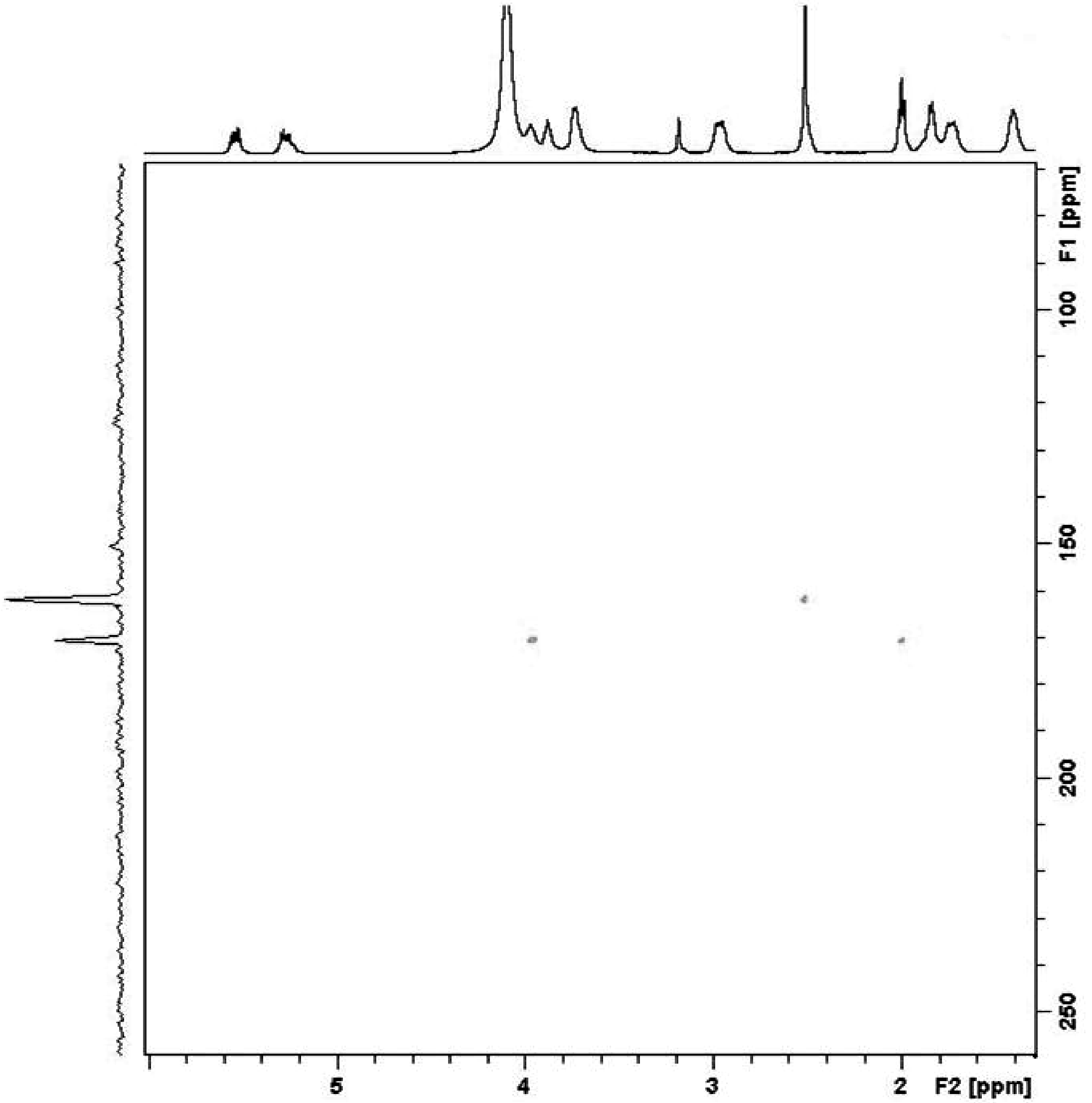

Figure S18. HR-ESI-MS spectrum of **3**.

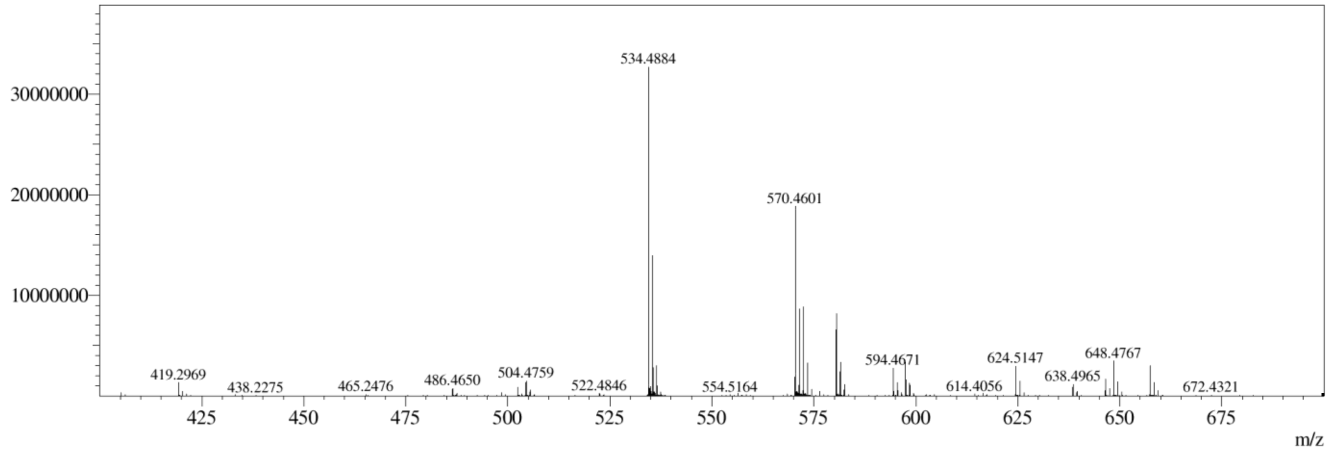

Figure S19. ^{1}H-NMR spectrum (500 MHz, pyridine-d_5) of **3**.

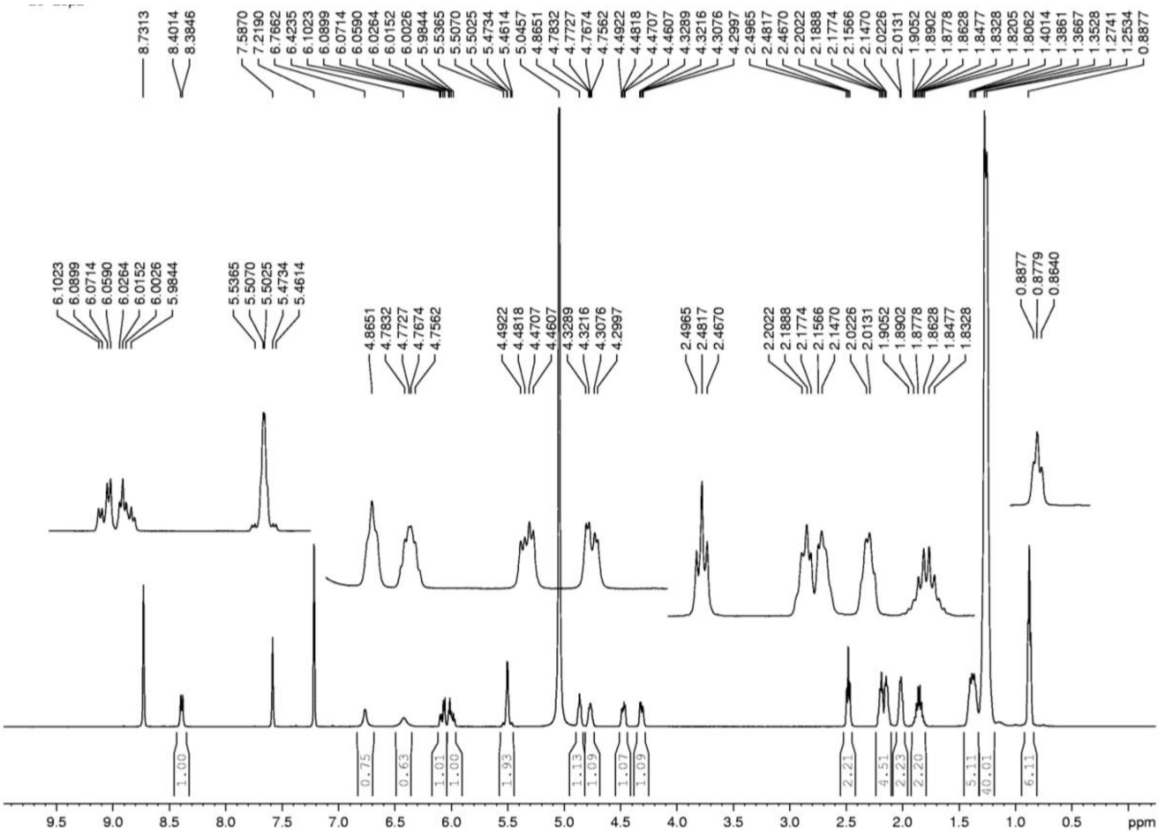

Figure S20. ^{13}C-NMR spectrum (125 MHz, pyridine-d_5) of **3**.

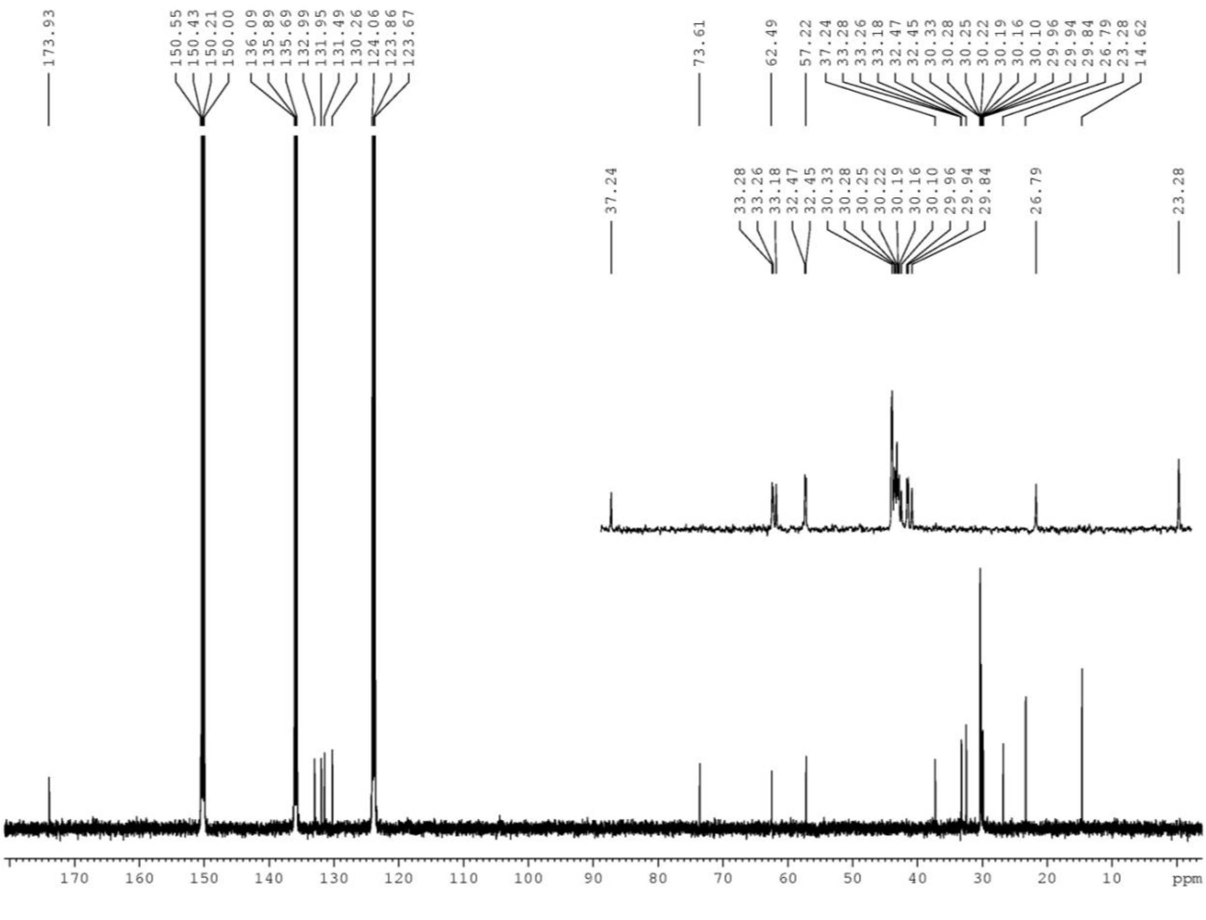

Figure S21. ^{1}H–^{1}H-COSY spectrum (500 × 500 MHz, pyridine-d_5) of **3**.

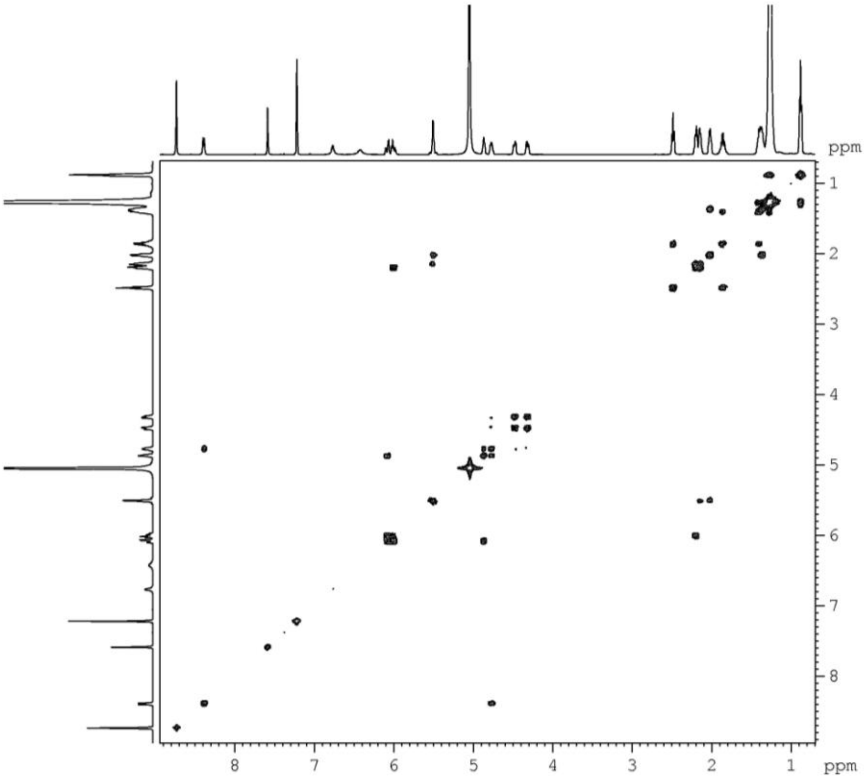

Figure S22. ^{1}H–^{13}C-HSQC spectrum (500 × 125 MHz, pyridine-d_5) of **3**.

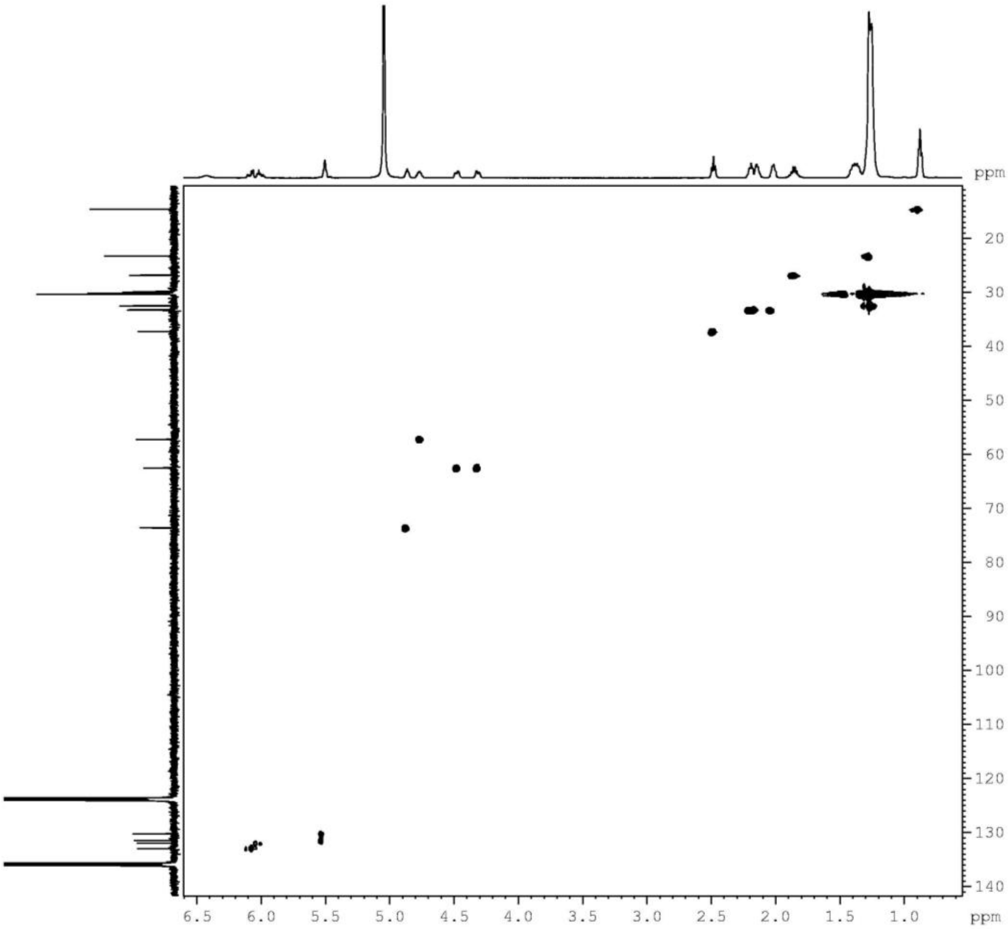

Figure S23. ^{1}H–^{13}C-HMBC spectrum (500 × 125 MHz, pyridine-d_5) of **3**.

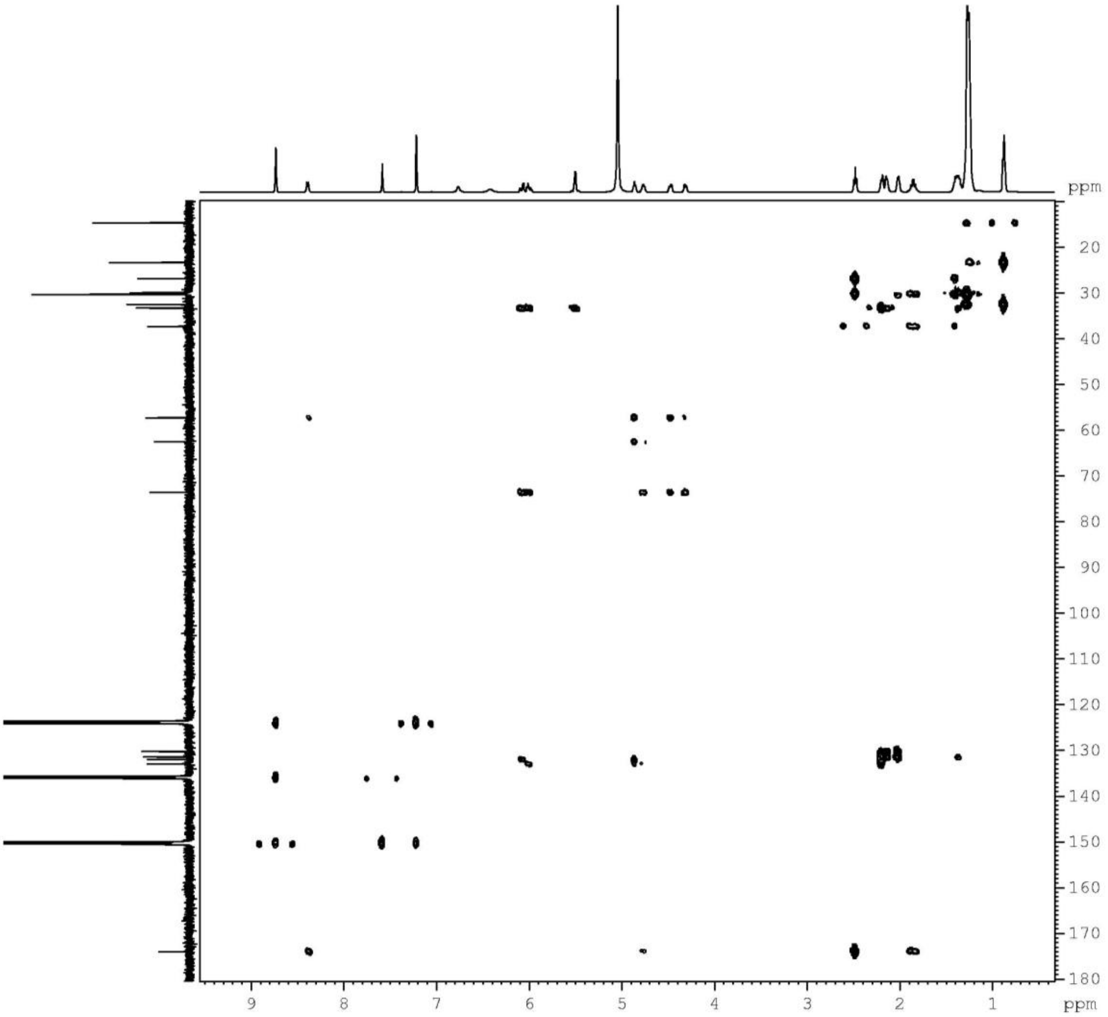

Figure S24. HR-ESI-MS spectrum of **4**.

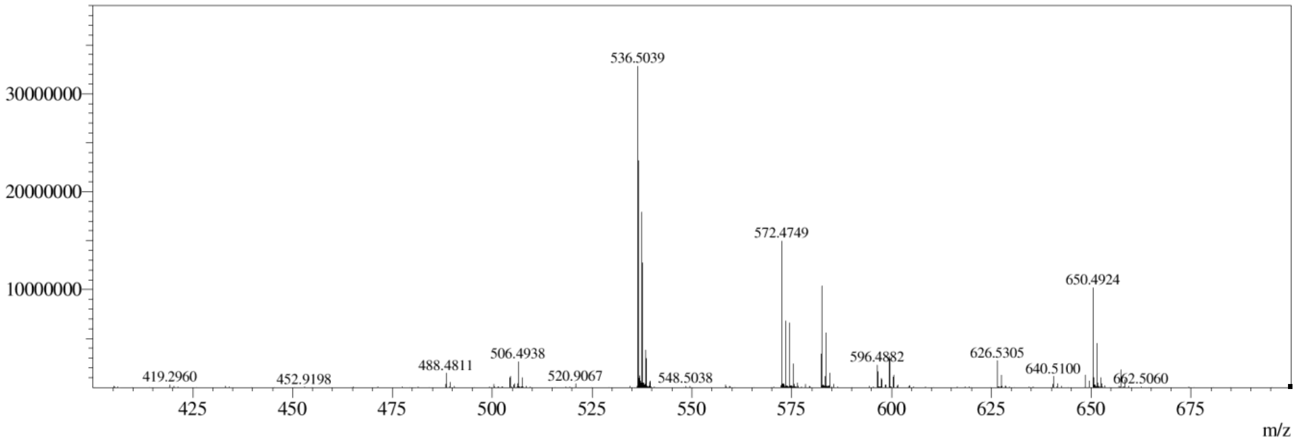

Figure S25. ^{1}H-NMR spectrum (500 MHz, pyridine-d_5) of **4**.

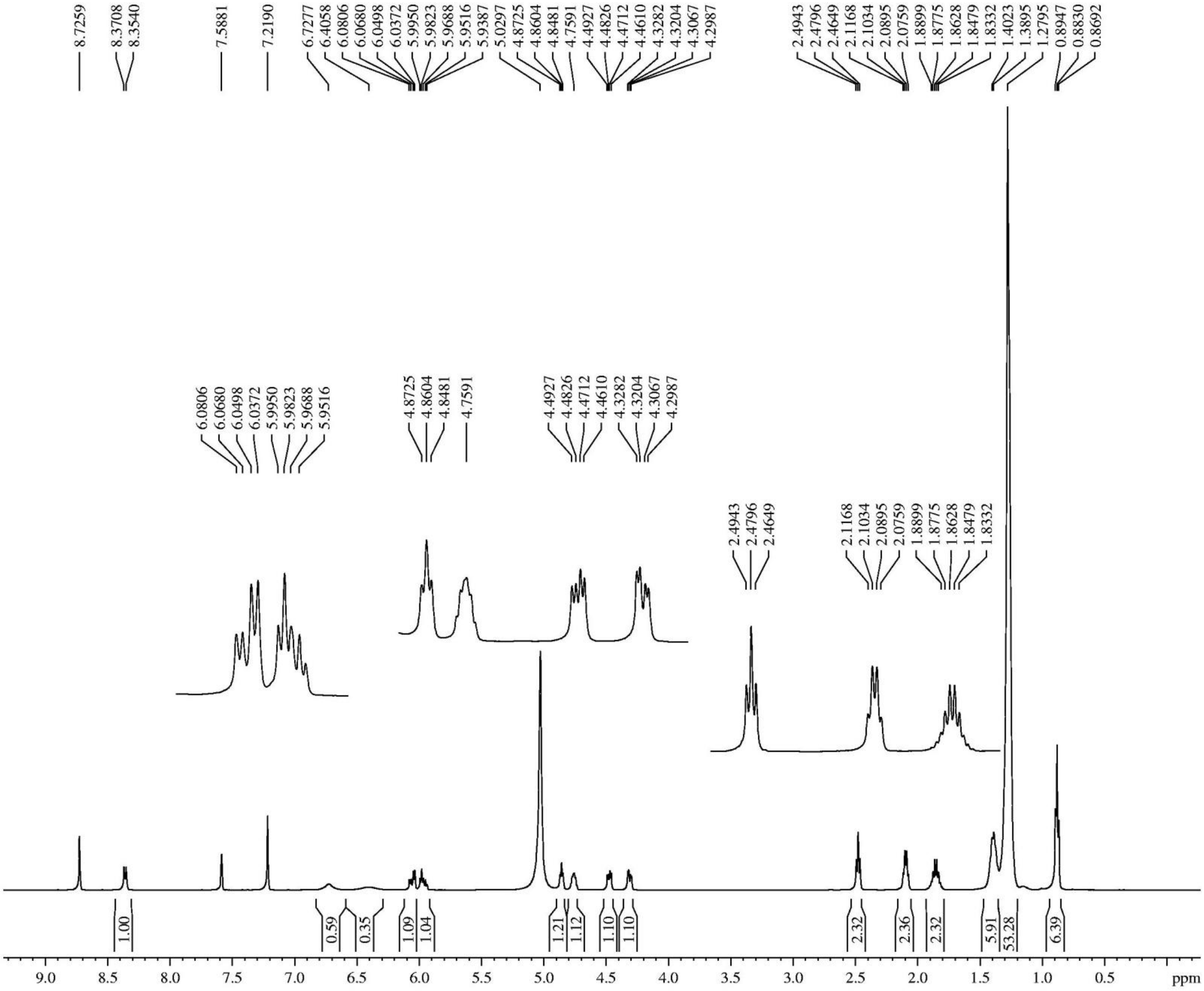

Figure S26. ^{13}C-NMR spectrum (125 MHz, pyridine-d_5) of **4**.

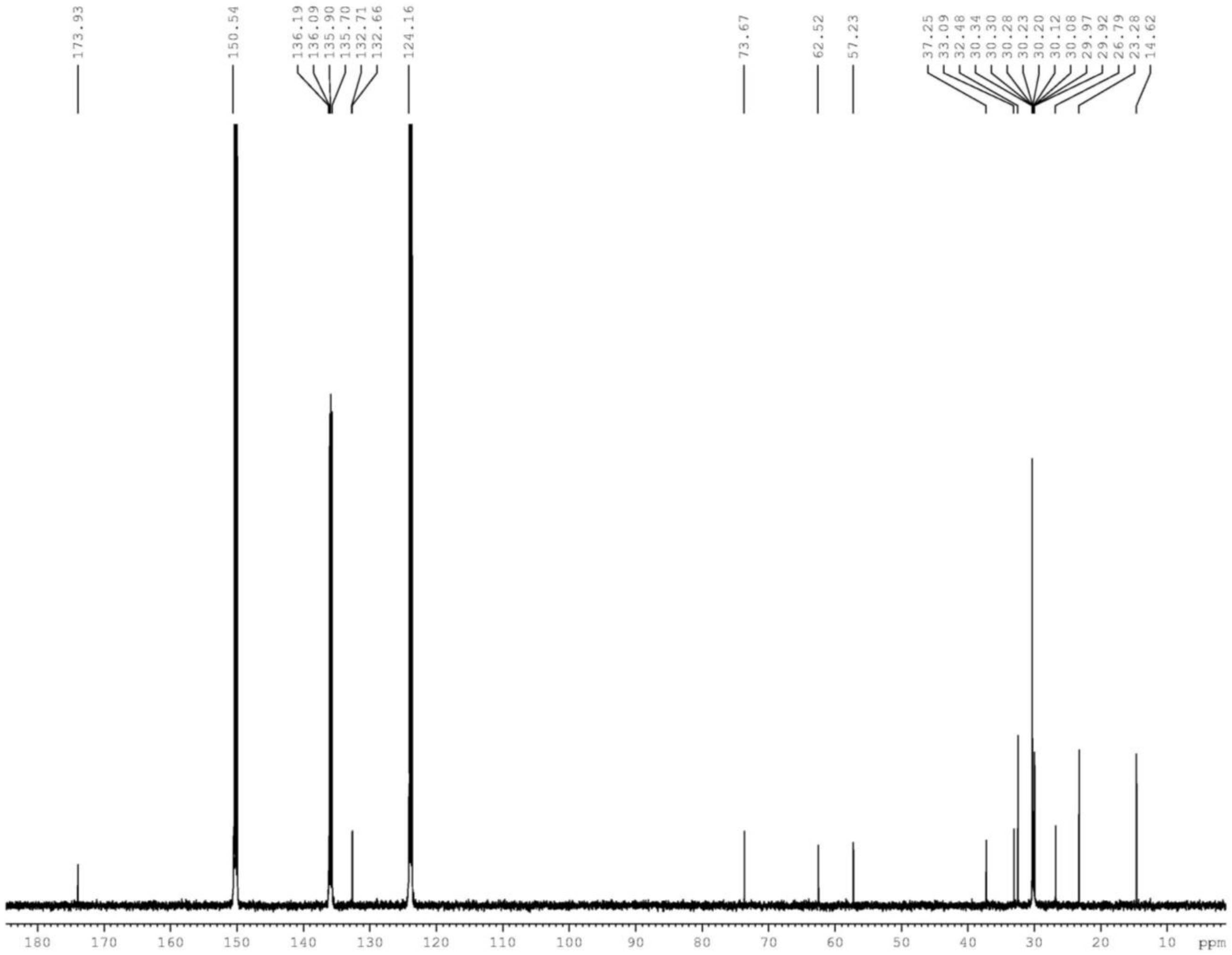

Figure S27. ^{1}H–^{1}H-COSY spectrum (500 × 500 MHz, pyridine-d_5) of **4**.

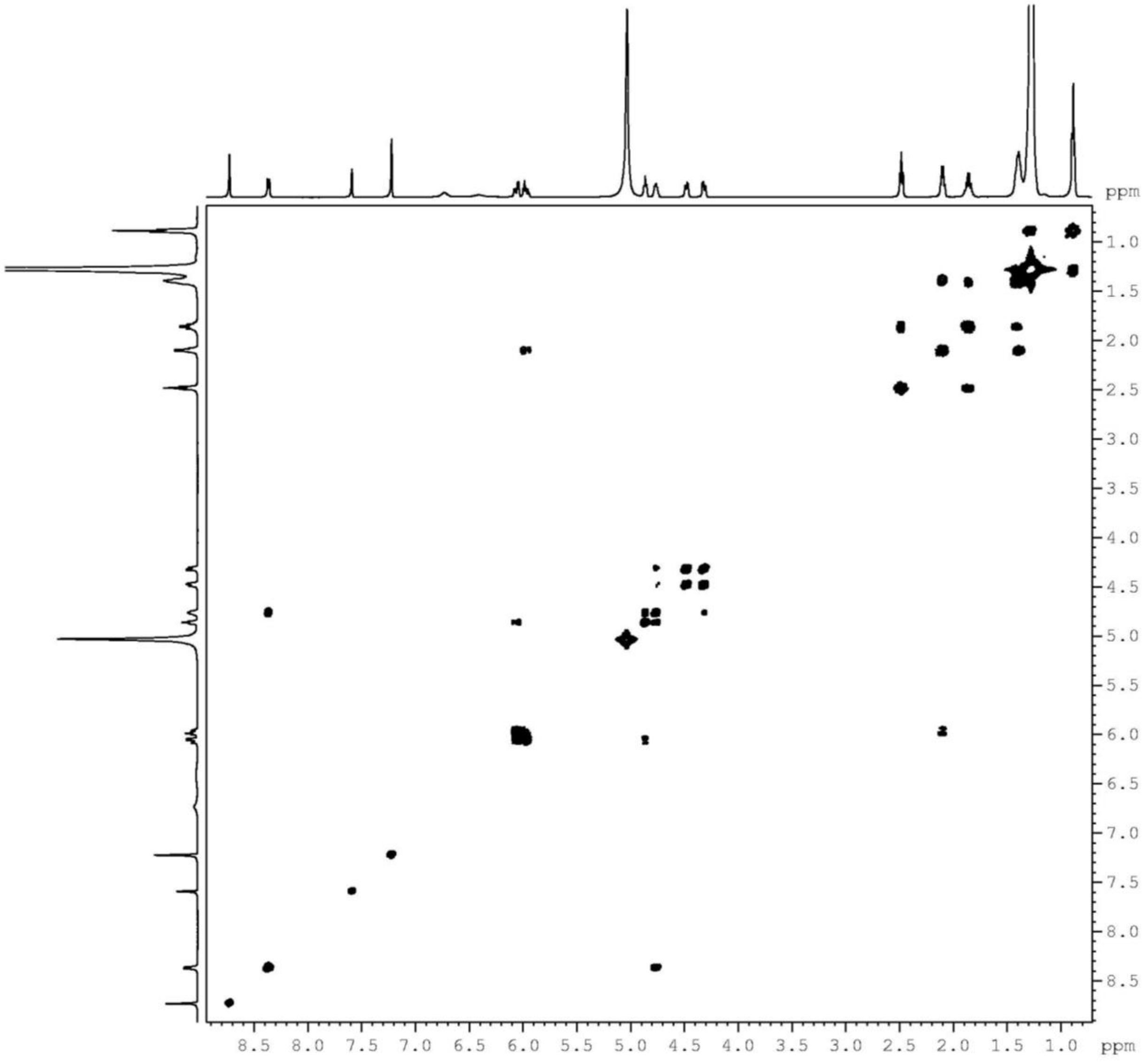

Figure S28. ^{1}H–^{13}C-HSQC spectrum (500 × 125 MHz, pyridine-d_5) of **4**.

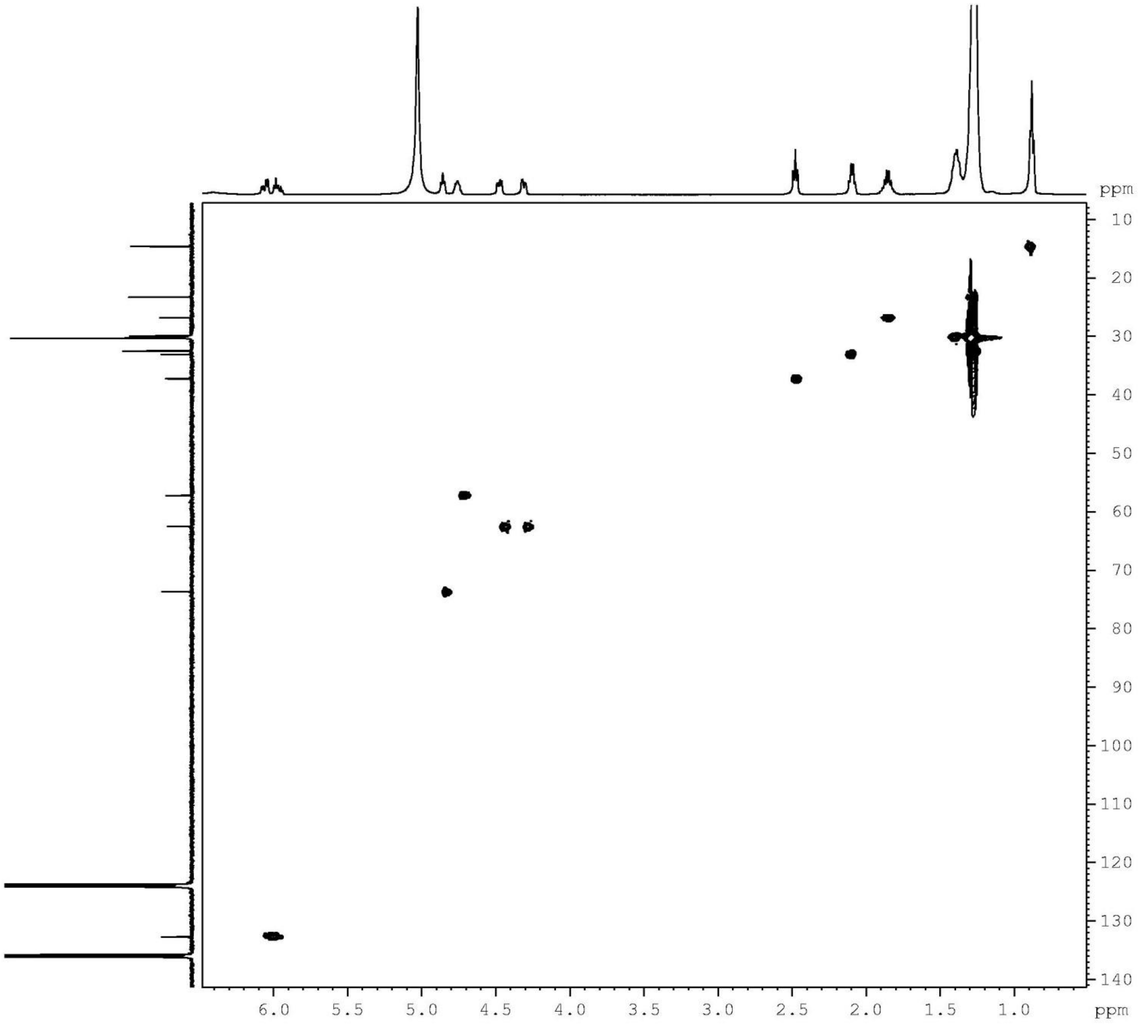

Figure S29. ^{1}H–^{13}C-HMBC spectrum (500 × 125 MHz, pyridine-d_5) of **4**.

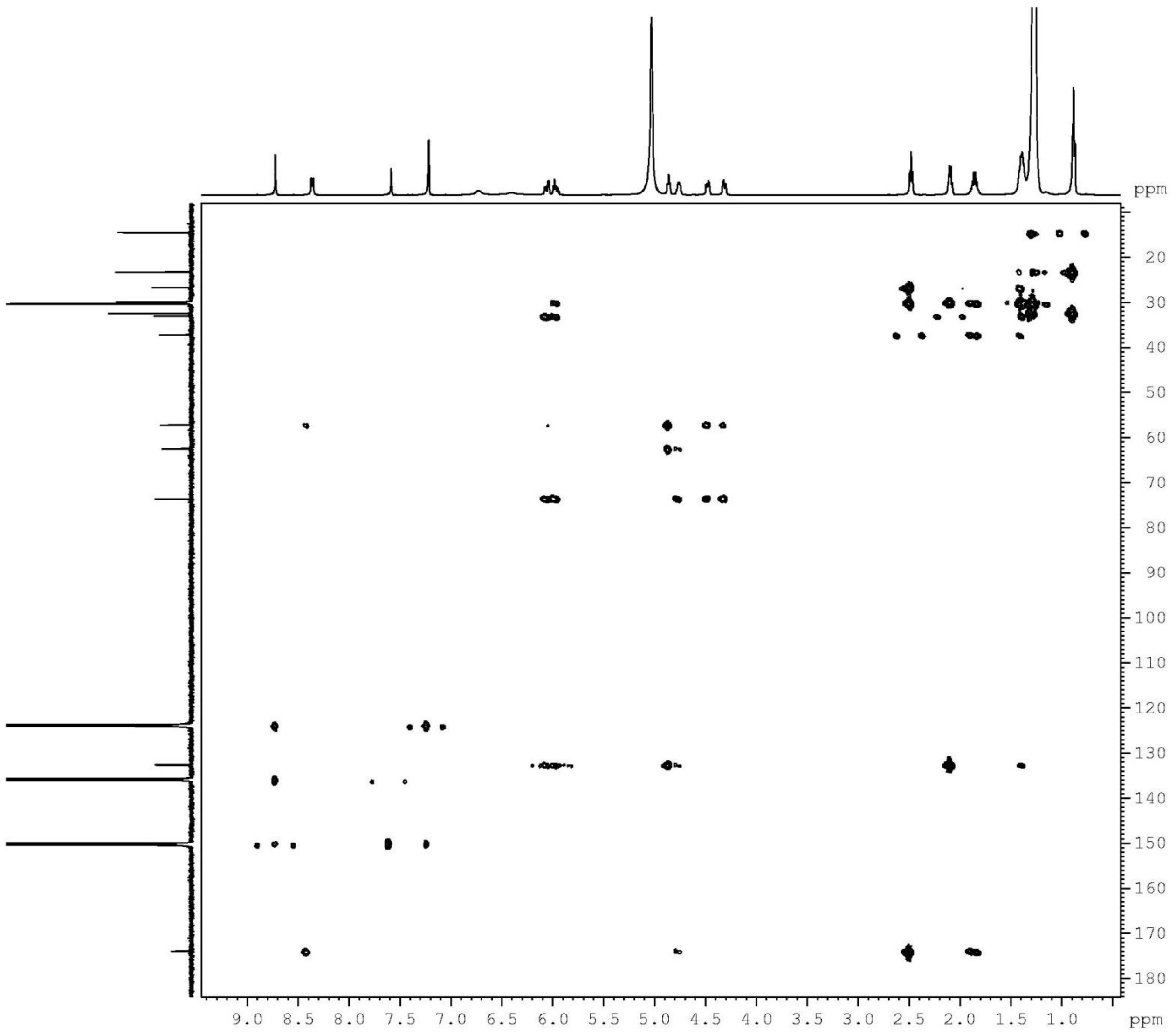

Figure S30. ^{1}H NMR spectrum (500 MHz, CDCL$_3$/CD$_3$OD) of **3**.

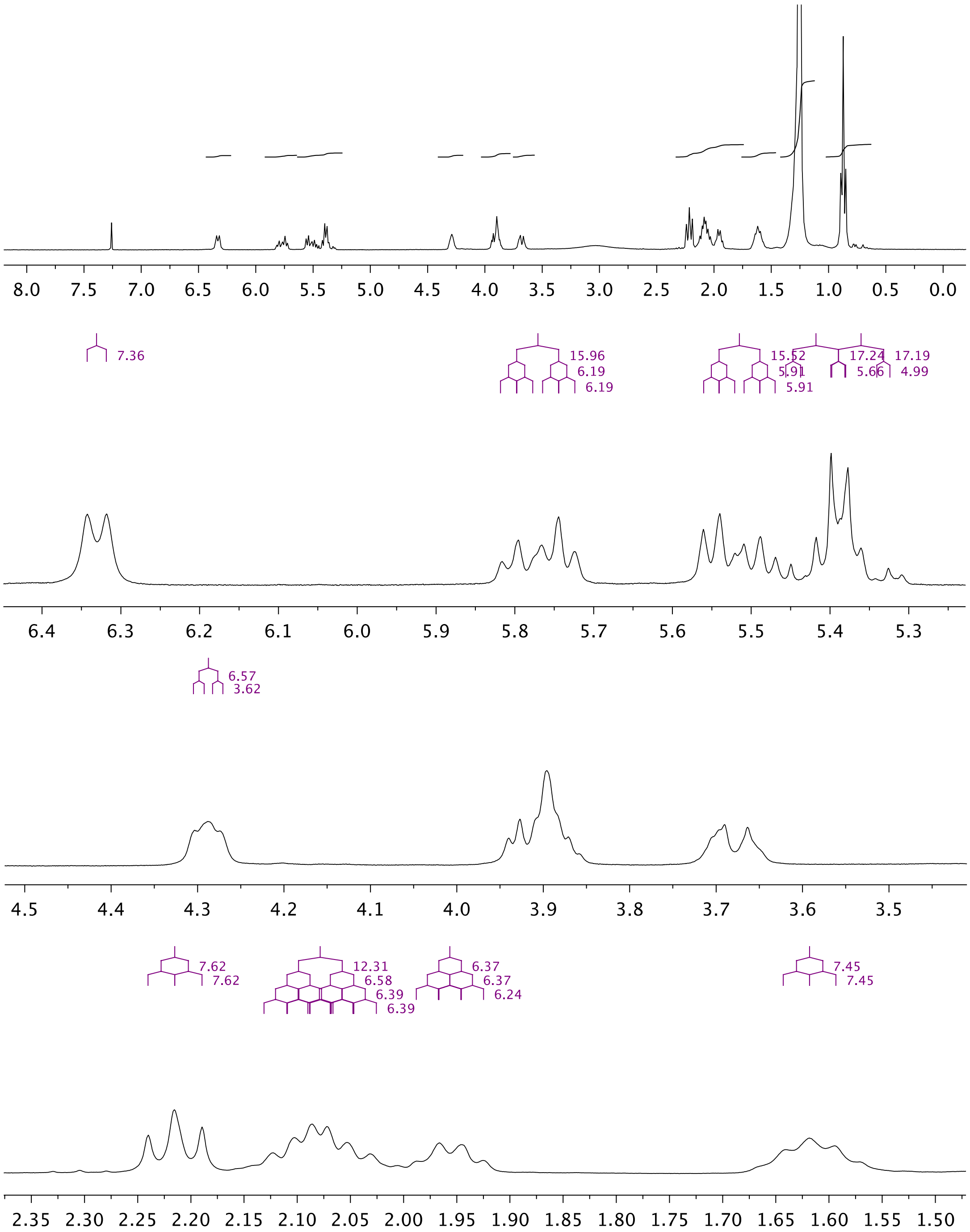

Figure S31. ^{13}C NMR spectrum (125 MHz, CDCL$_3$) of **3**.

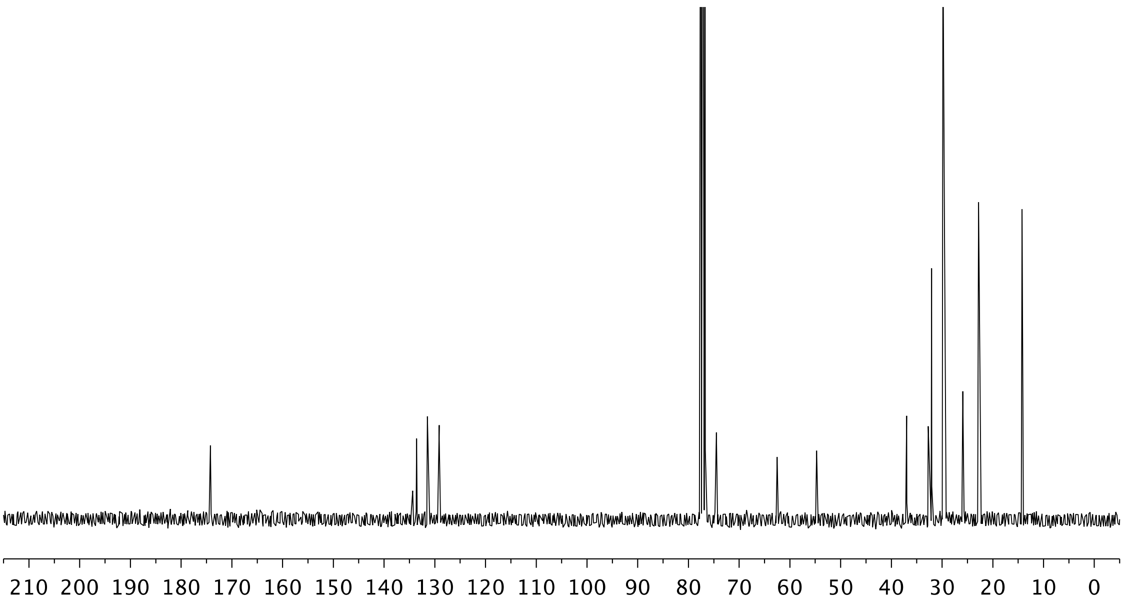

Figure S32. ^{1}H–^{13}C HSQC spectrum (500 × 125 MHz, CDCl$_3$) of **3**.

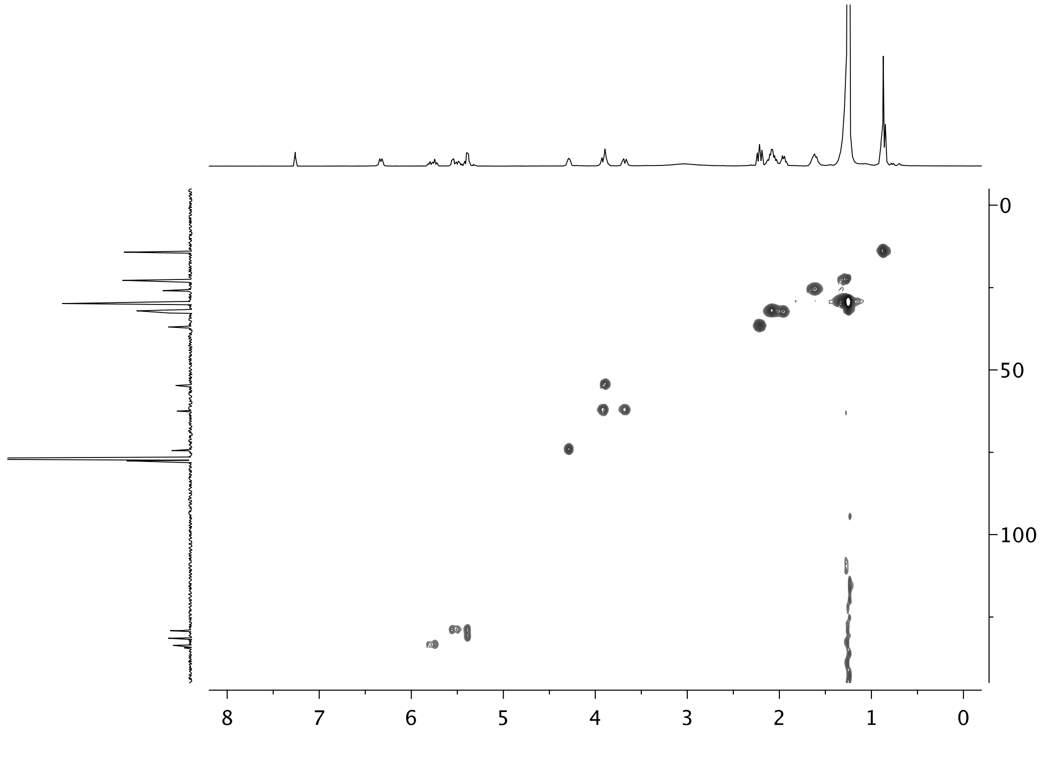

Figure S33. ^{1}H NMR spectrum (500 MHz, $CDCl_3$) of **4**.

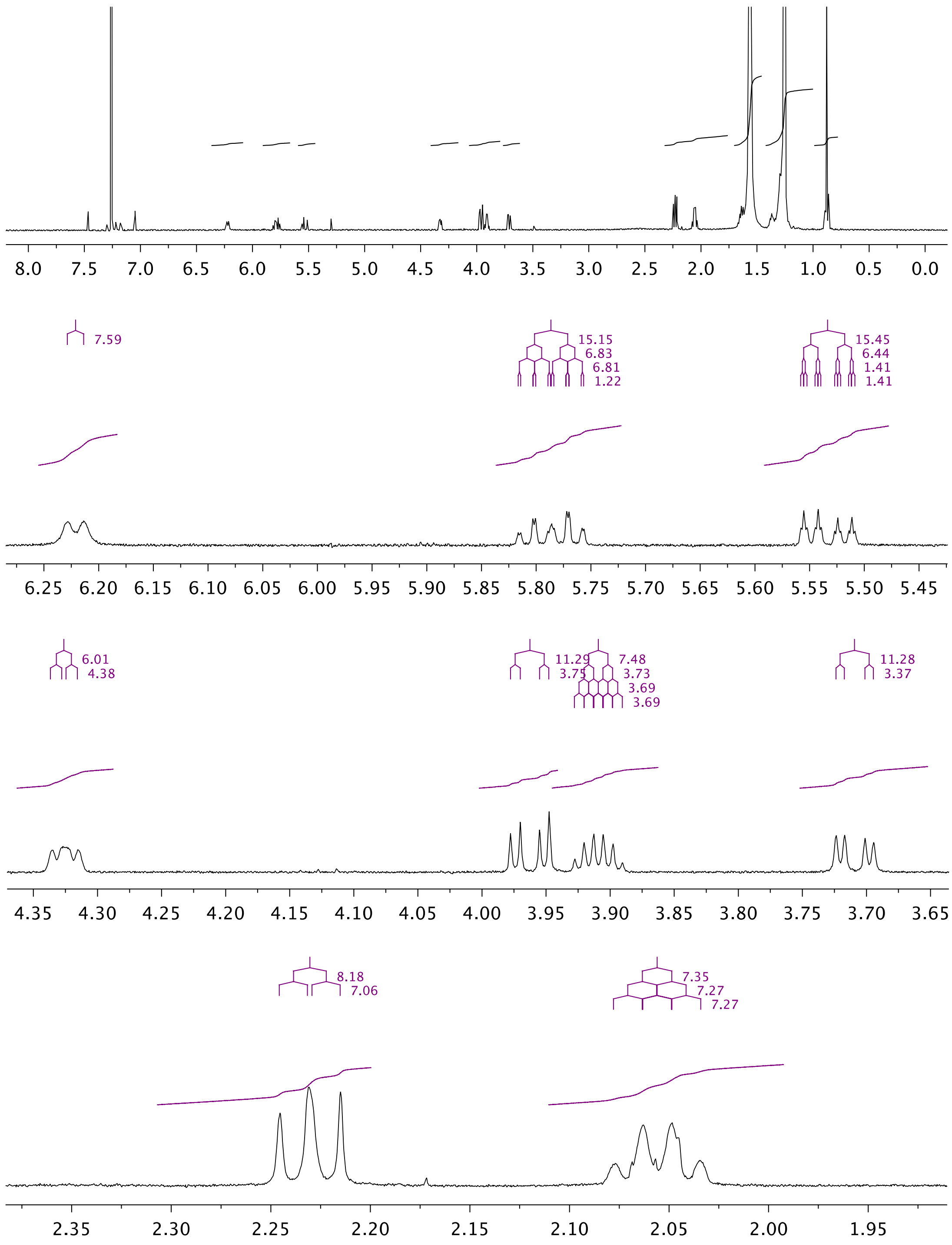

Figure S34. ^{13}C NMR spectrum (125 MHz, $CDCl_3$) of **4**.

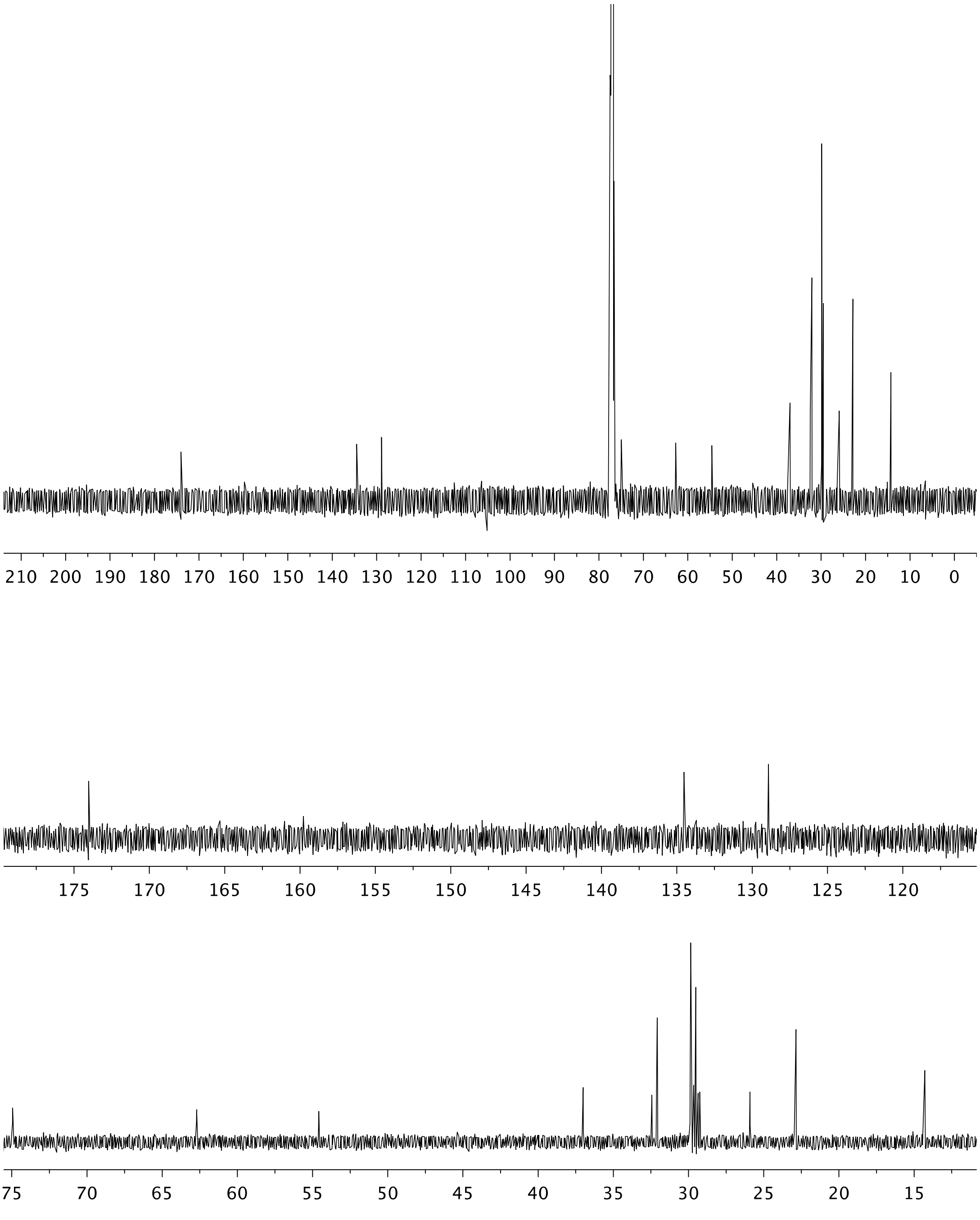

Supplement: Supplementary File 1 — Supplementary Information (PDF, 2609 KB) [file marinedrugs-10-02846-s001.pdf]
